# Microbiota-derived acetate is associated with functionally optimal virus-specific CD8^+^ T cell responses to influenza virus infection via GPR43-dependent metabolic reprogramming

**DOI:** 10.1080/19490976.2024.2401649

**Published:** 2024-10-10

**Authors:** Jingjing Qiu, Chunwei Shi, Yanan Zhang, Tianming Niu, Shuxian Chen, Guilian Yang, Shu Jeffrey Zhu, Chunfeng Wang

**Affiliations:** a College of Veterinary Medicine, Jilin Agricultural University, Changchun, P. R. China; b Engineering Research Center of Microecological Vaccines (Drugs) for Major Animal Diseases, Ministry of Education, Jilin Agricultural University, Changchun, P. R. China; c Department of Veterinary Medicine, College of Animal Sciences, Zhejiang University, Hangzhou, Zhejiang, P. R. China

**Keywords:** *Blautia coccoides*, acetate, virus-specific CD8^+^ T cells, influenza virus, GPR43, metabolic reprogramming

## Abstract

The microbiota-associated factors that affect host susceptibility and adaptive immunity to influenza A virus (IAV) infection have not been fully elucidated. By comparing the microbiota composition between survivors and mice that succumbed to IAV strain PR8 infection, we identified that the commensal bacterium *Blautia coccoides* protects antibiotics (Abx)-treated or germ-free (GF) mice from PR8 infection by inducing functionally optimal virus-specific CD8^+^ T cell responses. Administration of exogenous acetate reproduced the protective effect of *B. coccoides* monocolonization in Abx and GF mice, enhancing oxidative phosphorylation and glycolysis as well as secretion of IFN-γ and granzyme B in virus-specific CD8^+^ T cells, dependent on GPR43 signaling and acetyl-CoA synthetase 2. Thus, we have demonstrated that microbiota-derived acetate possesses an antiviral effect that induces an optimal virus-specific CD8^+^ T cell response to IAV PR8 infection via GPR43-dependent metabolic reprogramming.

## GRAPHICAL ABSTRACT

**Figure:**
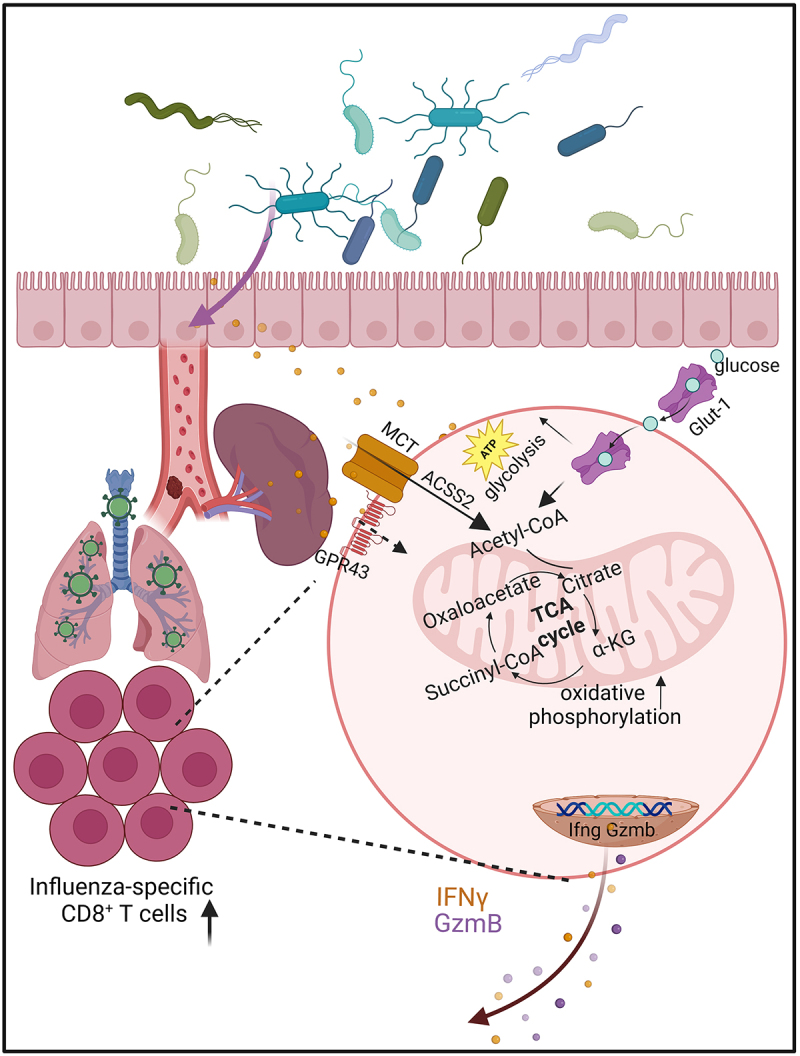

## Introduction

The mammalian gastrointestinal tract is colonized by an extensive, diverse and critically important population of microorganisms (termed the ‘microbiota’), which is known to perform biological functions including carbohydrate fermentation and biosynthesis of vitamins, hormones and neurotransmitters^1^. The gut microbiota generates immensely diverse bioactive small-molecule metabolites that modulate host physiology and immunity for maintenance of homeostasis.^2,3^ As such, microbiota dysbiosis has been linked not only to a variety of metabolic and inflammatory disorders but also to greater susceptibility to viral infections.^4^ In studies of patients and animal models, the gut microbiome during infection, the health status present, susceptibility, and clinical outcomes are linked. Therefore, the association of microbial differences, susceptibility and clinical symptoms in the dead and surviving groups was obtained.^5^

Influenza is an acute communicable respiratory illness that affects the upper and lower respiratory routes and is a severe threat to human health.^6–8^ Annually, influenza epidemics affect approximately 1 billion individuals, with 3–5 million severe cases and up to 500,000 fatalities. The heterogeneity of host susceptibility to influenza A virus (IAV) infection is increasingly attributed to variations in the gut microbiota.^9–11^ However, the role of specific microbes and the metabolites they generate in modulating IAV pathogenesis and disease outcome remain largely unknown.

It is increasingly apparent that gut commensal bacteria can influence innate immunity to IAV infection through microbial metabolites.^12–15^ For instance, a flavonoid degradation product processed by *Clostridium orbiscindens*, desaminotyrosine (DAT), has been shown to augment type I interferon (IFN-I) signaling in pulmonary macrophages, thereby protecting mice from IAV infection.^14^ Zhang and colleagues reported that *Bifidobacterium animalis* mediates a protective effect against avian influenza virus H7N9 by promoting type I and II IFN responses and ameliorating proinflammatory cytokines in the lung of infected mice through the biosynthesis of valine and CoA.^16^ However, the gut-lung axis crosstalk that influences host adaptive immunity at pulmonary mucosal sites and how this response impacts IAV pathogenesis are less well defined.

A functional role of the gut microbiome in regulating the CD8^+^ T cell response has been described. Diminished CD8^+^ T cell responses following infection of antibiotics (Abx)-treated or germ-free (GF) mice with IAV are associated with increased in vivo infection or viral susceptibility.^13,17^ It has been documented that short-chain fatty acids (SCFAs), especially butyrate which is fermented from dietary fiber by gut microbiota, confer protection against IAV by improving the cellular metabolism of CD8^+^ T cells via FFAR3 and fatty acid oxidation.^18^ Nevertheless, the identification of certain microbiome species and a mechanistic understanding of the signaling pathways involving in CD8^+^ T cell response and microbial metabolites in IAV infection are still lacking.

Here, by screening the intestinal microbiota of surviving vs dying mice following IAV infection, we identified a commensal bacterium, *Blautia coccoides*, that plays a crucial role in protecting mice from fetal IAV infection. Colonization of Abx or GF mice with *B. coccoides* restored the impaired virus-specific CD8^+^ T cell response attributed to microbiota depletion and significantly alleviated disease phenotypes after IAV infection. Administration of exogenous acetate reproduced the protective effect of *B. coccoides* in Abx and GF mice, rescuing suboptimal CD8^+^ T cell response in the lung and spleen in a G protein-coupled receptor 43 (GPR43)-dependent manner. Mechanistically, acetate boosted both oxidative phosphorylation (OXPHOS) and glycolysis and promoted secretion of IFN-γ and granzyme B in virus-specific CD8^+^ T cells that was dependent on acetyl-CoA synthetase 2 (ACSS2). Thus, we demonstrated that an acetate-producing commensal bacterium *B. coccoides* protects against severe IAV infection, and the acetate-mediated protection correlates with an enhanced virus-specific CD8^+^ T cell response via GPR43 signaling and metabolic reprogramming.

## Results

### Commensal microbiota from surviving mice enhances host defense against IAV PR8 infection in WT mice

**Figure 1:**
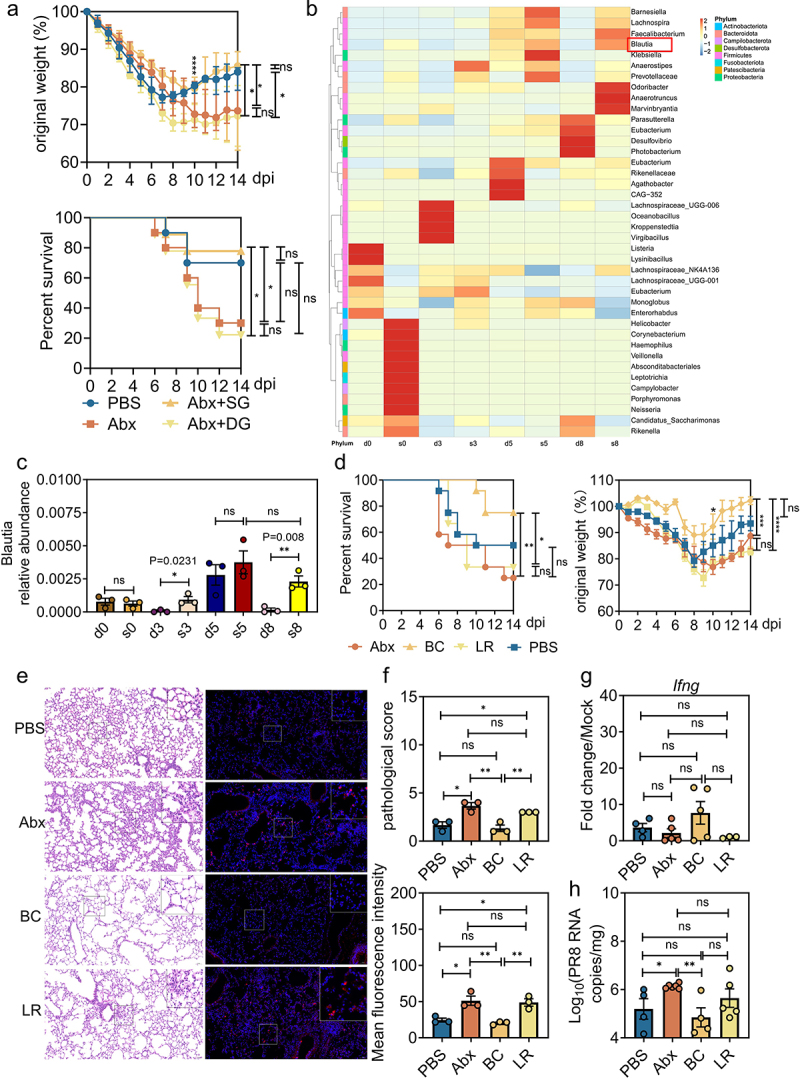
Commensal microbiota from surviving mice increases resistance to IAV infection in WT mice.

In a preliminary experiment, 30% of WT B6 mice infected by IAV H1N1 strain PR8 succumbed by the end of the 14-day observation period (Figure 1(a)). To investigate whether the observed heterogeneity in host susceptibility to IAV PR8 infection was associated with the intestinal microbiota, we pooled fecal samples collected from surviving (SG) or dying mice (DG) at 8 dpi and performed fecal microbiota transplantation (FMT) into mice whose microbiota was depleted by pretreatment with a cocktail of broad-spectrum antibiotics (Abx), as previously described.^19^ Similar to previously published literatures,^16,17^ Abx-treated, IAV PR8-infected mice lost significantly more body weight and had increased mortality compared to the phosphate-buffered saline (PBS)-treated, IAV PR8-infected controls, especially after 10 days post-infection (Figure 1(a)). Intriguingly, FMT from the SG fecal pool into Abx-treated mice significantly alleviated their weight loss and reduced mortality rate by approximately 40% following IAV PR8 infection, whereas Abx mice that received FMT from the DG fecal pool did not have changes in weight loss or mortality (Figure 1(a)). This indicated that the gut microbiome of surviving IAV PR8-infected mice may indeed contain specific bacterial species that were protective from lethal IAV infection.

a, Survival kinetics analysis and weight loss of PBS, Abx and Abx-treated FMT recipient mice (mice that received FMT from mice who survived or died following IAV PR8 infection) (*n* = 10). SPF C57BL/6 mice were nasally infected with IAV H1N1 strain PR8 (*n* = 10, 40 μL/mouse). b, Heatmap of the relative abundance of bacteria in feces from PR8-infected mice (*n* = 3 to 4). c, Relative abundances of bacterial genus in the surviving group (s0, s3, s5, s8) and dying group (d0, d3, d5, d8) (*n* = 3). d-g, d, Survival curve and weight loss of PBS-treated, Abx-treated, BC- or LR-colonized Abx mice following PR8 infection (*n* = 10 to 14, 40 μL/mouse). e, Left, pulmonary histology (magnification, 20×); scale bar, 50 μm. Right, immunofluorescence analysis of lung IAV staining of hemagglutinin (red) and nuclei (blue). f, Upper, blinded sections were assessed to determine the level of pathological severity. In order to evaluate the overall histological changes, lung tissue sections were scored according to the criteria specified in the panel. The scoring system used was as follows: 0, no pathological change; 1, affected area (≤20%); 2, affected area (≤50%, >20%); 3, affected area (>50%, ≤80%); 4, affected area (>80%). Lower, quantitative analysis of the fluorescence intensity of HA in lung, showing the mean fluorescence intensity. g, Relative *Ifng* expression in the lung (*n* = 3 to 5). h, Viral titers in lung collected from differently treated mice at 8 dpi following IAV PR8 infection determined by qRT-PCR (*n* = 4 to 6). Plotted data represent the mean ± s.d; unpaired two-tailed Student’s *t*-tests were used to compare means between treatment groups. *, *p* < 0.05; **, *p* < 0.01; ***, *p* < 0.001; ****, *p* < 0.0001; ns, not significant.

We next performed 16S rRNA gene sequencing (16S Group) on fecal samples collected from SG and DG mice at the indicated timepoints (Figure S1a). There was a remarkable difference in the bacterial taxonomic composition in the gut microbiota between SG mice and those who eventually succumbed to the viral infection (DG mice; Figure S1b). We observed a significantly greater abundance of *Blautia*, *Faecalibacterium* and *Barnesiella* in the SG mice compared with the DG mice on the species abundance clustering heat map at the genus level (Figure 1(b)). Consistently, there was a significantly increased relative abundance of *Blautia* in the survived vs succumbed IAV PR8-infected mice at 3 and 8 dpi in the histogram of species abundance (Figure 1(c), Figure S1d). In agreement with this phenotype, we observed both significantly higher relative abundance of *Blautia* in the Abx-SG mice than the Abx-DG mice by 16S rRNA sequencing (Figure S1c). We chose *B. coccoides* (abbreviated as BC hereafter), an anaerobic Gram-positive commensal bacterial strain that has been reported it can promote macrophage activation and I IFN responses in mononuclear phagocytes to limit EMCV infection,^19^ as representative isolates to determine how they protect against IAV PR8 infection. Further qPCR analysis of fecal microbiota revealed a significantly higher level of this particular bacterial strain in the Abx recipient mice receiving microbiota from SG mice compared to those receiving microbiota from the DG group (Figure S1e).

To further determine the role of BC in protecting mice against IAV infection, groups of Abx-treated WT B6 mice (abbreviated as Abx hereafter) were colonized with BC (abbreviated as BC hereafter) for 48 h and then nasally inoculated with IAV PR8. A well-studied human symbiotic isolate *Lactobacillus reuteri* (abbreviated as LR hereafter) served as an unrelated Gram-positive commensal bacterium control. Although both BC and LR efficiently repopulated the gut microbiota of Abx-treated mice (Figure S1f, Figure S1g), colonization by BC, but not LR, significantly reduced body weight loss and increased the survival rate from 20% to 80% by the end of the observation period (Figure 1d). In addition, reconstitution of gut microbiota by BC, but not LR, in Abx-treated mice exerted a probiotic effect in ameliorating tissue damage and reducing HA proteins, as reflected by pathological score and mean fluorescence intensity (Figure 1(e,f)). Also, there was increased expression of the antiviral cytokine IFN-γ in the lung upon IAV PR8 infection at 8 dpi, although the difference was not statistically significant (Figure 1(g)) and reduction in viral genomic copies (Figure 1(h)). These findings suggest a specific role for BC in strengthening IAV clearance and alleviating immunopathological changes in pulmonary tissues.

### B. coccoides *colonization enhanced innate antiviral immune responses to IAV PR8 infection*

**Figure 2:**
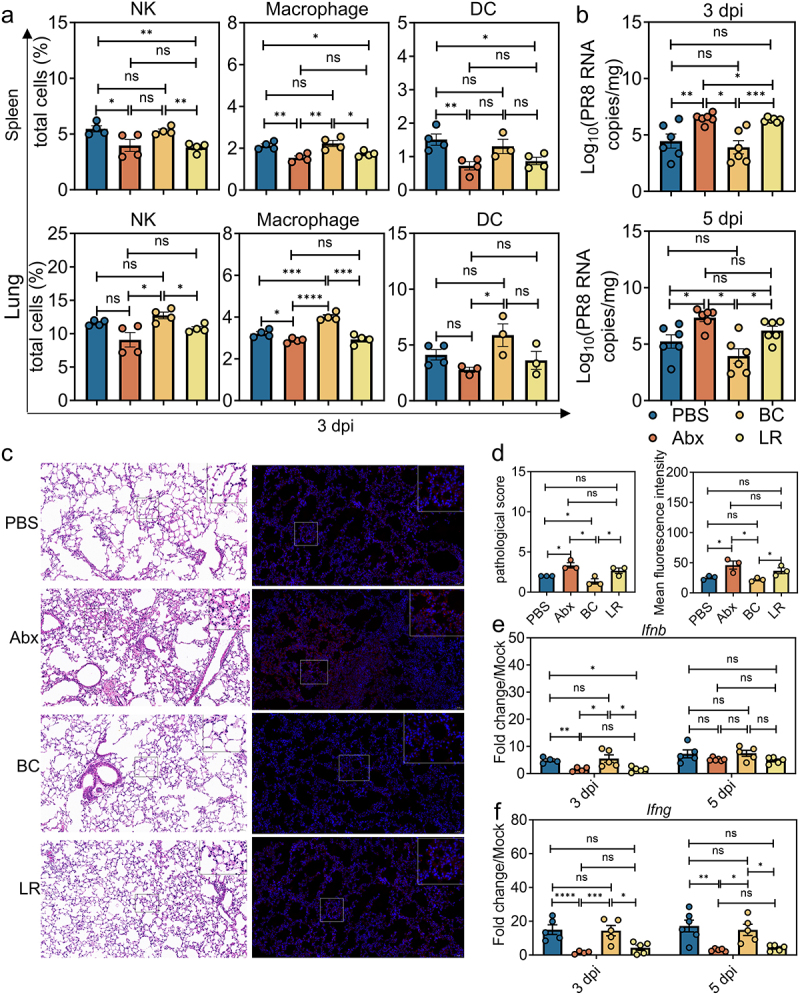
Innate cellular immune responses after IAV PR8 infection were greatly diminished in Abx-treated mice.

Since we have previously demonstrated that BC can limit EMCV infection systemically by priming IFN-I responses in peripheral mononuclear phagocytes,^19^ we first tested the recruitment and activation of early responding innate immune cells in the lung and spleen of noncolonized, BC- or LR-colonized Abx mice at 3 dpi of IAV PR8 infection by flow cytometry. In addition to this, we showed the process of flow cytometric circle gating of relevant immune cells (Figure S2a). There was a significant increase of natural killer (NK) cells, macrophages and dendritic (DC) cells in the lung of Abx mice colonized with BC, but not LR compared with the uncolonized, IAV PR8-infected control animals (Figure 2(a)). There was also a trend for BC colonization in upregulating the frequency of NK cells and DCs in the spleen following PR8 infection though the difference was not statistically significant (Figure 2(a)). In contrast, neither BC nor LR colonization altered the frequencies of pulmonary or splenic CD4 or CD8^+^ T or B cells at 3 dpi in the context of IAV PR8 infection (Figure S2b and S2c).

a, Frequency of a panel of innate immune cells, including natural killer (NK) cells, mononuclear phagocytes and dendritic cells (DC), in spleen or lung of PBS-treated, Abx-treated, BC- or LR-colonized Abx mice at 3 dpi (*n* = 3 to 4). b, Viral titers in lung collected from differently treated mice at 3 or 5 dpi following IAV PR8 infection (*n* = 6). c, Left, pulmonary histology (magnification, 20×); scale bar, 50 μm. Right, immunofluorescence analysis of lung IAV staining of hemagglutinin (red) and nuclei (blue). d, Left, blinded sections were assessed to determine the level of pathological severity. In order to evaluate the overall histological changes, lung tissue sections were scored according to the criteria specified in the panel. The scoring system used was as follows: 0, no pathological change; 1, affected area (≤20%); 2, affected area (≤50%, >20%); 3, affected area (>50%, ≤80%); 4, affected area (>80%). Right, quantitative analysis of the fluorescence intensity of HA in lung, showing the mean fluorescence intensity. e, Relative *Ifnb* expression in the lung (*n* = 4 to 6). f, Relative *Ifng* expression in the lung (*n* = 4 to 6). Plotted data represent the mean ± s.d; unpaired two-tailed Student’s *t*-tests were used to compare means between treatment groups. *, *p* < 0.05; **, *p* < 0.01; ***, *p* < 0.001; ****, *p* < 0.0001; ns, not significant.

To test whether the activation of innate immune cells attributed to BC colonization also translated into early control of IAV PR8 infection, we detected viral replication in the lung of infected mice at the RNA and protein level at 3 and 5 dpi, using qRT-PCR and immunofluorescent assay (IFA), respectively. Indeed, IAV PR8 infection resulted in significantly greater titer in the lung of Abx-treated mice compared with the BC- or PBS-treated (PBS control mice that had never received Abx) mice (Figure 2(b–d)). Consistent with this phenotype, Abx mice colonized with BC (but not LR) had significantly higher expression of IFN-γ cytokines in the lungs, and there was a trend toward increased IFN-β in the lungs, but no difference at 5 dpi (Figure 2(e,f)). We also assessed the expression of the lung pro-inflammatory cytokine IL-6 by qRT-PCR and found that levels of this cytokine were increased in uncolonized Abx mice compared with BC-colonized mice. In addition to this, we assessed the expression of the pro-inflammatory cytokine IL-6 in the serum of mice by ELISA, finding it increased in uncolonized Abx mice compared to BC-colonized mice, but there was no difference at 3 dpi (Figure S2d). Consistently, BC-colonized mice had less severe tissue inflammatory infiltration and lesions than LR-colonized or uncolonized controls at 5 dpi. These data suggest that BC modulates host antiviral innate immunity by enhancing antiviral type I and type II IFN and reducing proinflammatory cytokine expression. In conclusion, Abx mice revealed a reduced capacity to control viral replication. Following IAV PR8 infection, inflammasome activation led to migration of dendritic cells from the lung to the draining lymph node and T cell priming. According to our experimental data, there was a significant increase of dendritic (DC) cells of lung in Abx mice colonized with BC, but not LR compared with the uncolonized, IAV PR8-infected control animals. In addition, we have described that the difference of BC content between the SG group and the DG group is greater at 8 dpi. As a result, we speculated that BC has a greater impact on adaptive immunity.

### B. coccoides *colonization rescued impaired virus-specific CD8^+^ T cell response to IAV PR8 infection*

**Figure 3:**
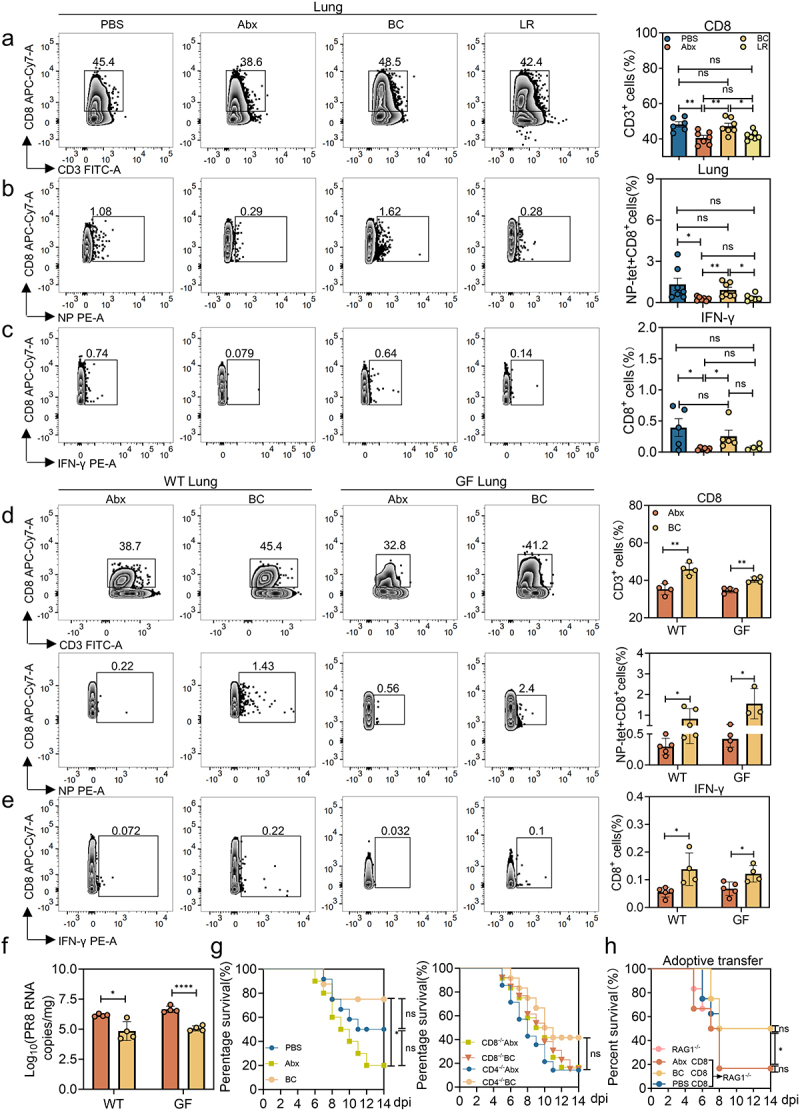
*B. coccoides* colonization boosted antiviral CD8^+^ T cell responses.

Given that BC colonization in Abx mice restored the capacity for IAV PR8 clearance at 8 dpi, we hypothesized that the adaptive antiviral responses were altered by BC. To test this, the proportion of T cell subsets were detected by Flow Cytometry (abbreviated as FCM hereafter) at 8 dpi. Interestingly, Abx treatment led to a significant reduction of CD8^+^, but not CD4^+^ T cell subsets in the spleen or lung, and BC colonization restored the percentage of CD8^+^ T cells in these tissues (Figure 3(a) and Figure S3a). Consistent with this finding, Abx WT mice colonized with BC generated significantly higher populations of virus-specific CD8^+^ T cells both in the lung and spleen upon IAV PR8 infection (Figure 3(b) and Figure S3b), although the overall frequencies of bronchoalveolar lavage fluid (abbreviated as BALF hereafter) CD4^+^ and CD8^+^ T cells were comparable among the different groups (Figure S3c). Additionally, CD8^+^ T cells in the spleen, lung and BALF of Abx mice were less capable of expressing activation marker (CD69) or producing multiple effector molecules including IFN-γ and granzyme B, whereas BC colonization restored expression of CD69 and secretion of IFN-γ and granzyme B in all three tissues upon IAV PR8 infection at 8 dpi (Figure 3(c) and Figure S3d). In agreement with the elevated expression of these molecules, the viral burden was greatly decreased in the BC-colonized Abx mice compared with the noncolonized (Figure S3e). We also tested the frequency of CD4^+^ and CD8^+^ T cells in the spleen of PBS, Abx, LR and BC mice at 12 dpi. There was an increased percentage of splenic CD8^+^ T cells in the noncolonized Abx mice compared with BC-colonized Abx mice or PBS control, although the differences were not statistically significant, implying a delay in the adaptive immune response to IAV and sustained activation of CD8^+^ T cells due to prolonged viral replication (Figure S3f).

a-c, PBS-treated, Abx-treated, BC- or LR-colonized Abx mice at 8 dpi after IAV PR8 infection. (a), Frequency of CD8^+^ T cells in the lung (*n* = 6 to 7). (b), Frequency of influenza nuclear protein (NP)-positive CD8^+^ T cells in the lung (*n* = 6 to 7). (c), FACS analysis of IFN-γ production by CD8^+^ T cells in the lung (*n* = 4 to 6). d-f, Abx-treated or BC-colonized, PR8-infected WT or GF mice at 8 dpi after IAV PR8 infection. (d), the pulmonary frequency of CD8^+^ T cells and NP-positive CD8^+^ T cells (*n* = 3 to 5). (e), FACS analysis of IFN-γ production by CD8^+^ T cells in the lung (*n* = 4 to 5). (f), Viral titers in the lung (*n* = 4). g, Survival curves of PBS-treated, Abx-treated, or BC-colonized WT mice (*n* = 10 to 12), and Abx-treated or BC-colonized CD4^−/−^ or CD8^−/−^ mice after IAV PR8 infection (*n* = 12 to 14). h, Survival of Rag1^−/−^ mice with or without adoptive transfers of CD8^+^ T cells collected from IAV PR8-infected WT mice treated with PBS, Abx, or BC at 8 dpi (*n* = 6 to 8). Plotted data represent the mean ± s.d.; unpaired two-tailed Student’s *t*-tests were used to compare means between treatment groups. *, *p* < 0.05; **, *p* < 0.01; ***, *p* < 0.001; ****, *p* < 0.0001; ns, not significant.

To exclude the possibility that the observed phenotypes were influenced by residual Abx-resistant microbes in the intestine of our Abx-treated mice, germ-free (GF) mice were colonized with BC, and the reconstitution of gut microbiota was confirmed by qPCR before IAV PR8 infection (Figure S3g). Consistent with the results observed in the Abx-treated mouse model, BC colonization resulted in a marked increase in both total and PR8-specific CD8^+^ T cells in the lung and spleen of GF mice, whereas total CD4^+^ T cells were not altered in either tissue of BC-colonized GF mice at 8 dpi (Figure 3(d) and Figure S3h). Further, CD8^+^ T cells in the lung and spleen of BC-colonized GF mice expressed significantly higher levels of CD69, IFN-γ and granzyme B compared with those from noncolonized GF mice following IAV PR8 infection at 8 dpi (Figure 3(e) and Figure S3i). Consistently, significantly greater titers of PR8 (Figure 3(f)), tissue damages (Figure S3j) and the pathological score (Figure S3k) were detected in the lung of noncolonized GF mice compared with the BC-colonized GF mice. Moreover, noncolonized GF mice exhibited more viral signals (immunofluorescence intensity of specific HA protein of influenza virus, hemagglutinin, abbreviated as HA hereafter) as well as the fluorescence density of HA in lung tissue than GF mice colonized by BC (Figure S3l).

Since BC colonization restored functional CD8^+^ T cell responses to IAV PR8 primary infection at the early phase of adaptive immunity, we next determined whether CD8^+^ T cells were required for the antiviral protective immunity mediated by BC. To this end, Abx-treated WT, CD4^−/−^ and CD8^−/−^ mice were colonized with BC and then inoculated with IAV PR8 and observed for mortality for 14 days. There was no significant difference in the relative weight loss or survival rates between noncolonized and BC-colonized Abx CD8^−/−^ mice, whereas the noncolonized Abx CD4^−/−^ mice had a significantly higher weight loss than BC-colonized Abx CD4^−/−^ mice. It also increased mortality rate, although there was no difference (Figure 3(g) and Figure S3m, S3n), indicating that CD8^+^ and not CD4^+^ T cells were required for BC-mediated protection from IAV PR8 at the early phase of activation of adaptive immunity.

To test whether CD8^+^ T cells are sufficient to mediate protection against IAV PR8 infection, we next carried out adoptive transfers of CD8^+^ T cells from different types of PR8-infected mice into naïve Rag1^−/−^ recipients, which lack mature T and B cells. To this end, we purified CD8^+^ T cells from the spleens of PR8-infected PBS, Abx or Abx-BC WT mice and adoptively transferred them into naïve Rag1^−/−^ recipients via tail vein injection. The purity of splenic CD8^+^ T cells and the efficiency of adoptive transfers were validated by flow cytometry (Figure S3o). According to our data, the transferred PR8-specific CD8^+^ T cells in the spleen collected from Abx- or BC-treated mice at 8 days post-IAV PR8 infection were detected at the average frequency of 0.189% (3,780) and 2.69% (53,800) of CD8^+^ T cells (2 × 10^6^ total transferred CD8^+^ T cells), respectively (Figure S3p). Rag1^−/−^ recipient mice were infected with IAV PR8 at 1 day-post-transfer and assessed for weight loss and mortality. Adoptive transfer of splenic CD8^+^ T cells from PBS- or BC-colonized Abx WT mice significantly increased survival and slowed weight loss compared to non-transferred controls, although the difference was not significant (Figure 3(h) and Figure S3q). On the contrary, Rag1^−/−^ recipients adoptively transferred with CD8^+^ T cells purified from spleens of noncolonized Abx WT mice conferred no protection against IAV PR8 infection (Figure 3(h)). Taken together, these data demonstrate that BC was capable of inducing significantly higher populations of virus-specific CD8^+^ T cells and enhancing the effector function of CD8^+^ T cells to antagonize IAV PR8 infection.

### *The* B. coccoides *metabolite acetate protects mice against IAV PR8 infection by inducing virus-specific CD8^+^ T cells*

**Figure 4:**
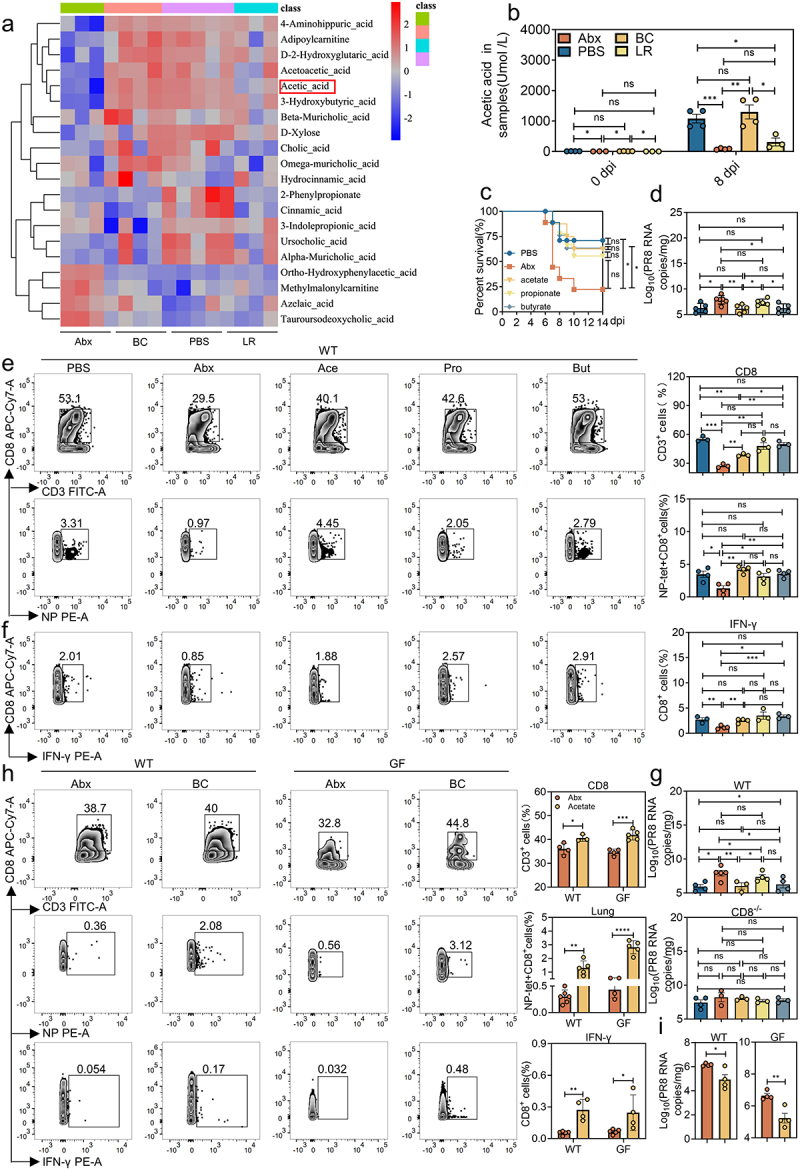
*B. coccoides* metabolite acetate rescued the generation of iav-specific CD8^+^ T cells.

Since the gut microbiota generates or modifies many bioactive small metabolites that diffuse into circulation to modulate host immune responses in extraintestinal tissues,^2,20^ we next performed untargeted metabolomics analysis on serum collected from PBS, Abx, Abx-BC and Abx-LR groups of mice at 0 and 8 days post-IAV PR8 infection to identify the metabolites involved in BC-related protection against IAV PR8 infection (Figure 4a and Figure S4a). The Pareto-scaled principal component analysis (PCA) demonstrated significant differences in metabolomics profiles of Abx-treated mice compared with those of PBS control or bacteria-colonized groups (Figure S4b). KEGG analyses revealed that the differential metabolites in PBS control mice (compared with Abx-treated mice) were mainly enriched in carbohydrate and amino acid metabolism (Figure S4c). Of particular note, the absolute serum concentration of acetate was significantly reduced in Abx mice and restored by BC, but not LR, colonization prior to and at 8 dpi (Figure 4(b)). Consistent with this finding, we analyzed the BC cultured supernatants in vitro with GC-MS/MS and identified high level of acetate among all the SCFAs (Figure S4d). In addition, there was a significantly higher serum acetate concentration in the Abx mice receiving SG fecal transplantation than the DG recipients (Figure S4e), demonstrating a positive correlation between the serum acetate concentration and resistance to IAV PR8 infection when Abx mice were transplanted with fecal microbiota from SG or DG mice.

a, Heatmaps show differences untargeted metabolomics analysis of PBS-treated, Abx-treated, BC- or LR-colonized Abx mice at 8 dpi after IAV PR8 infection (*n* = 3 to 5). b, Acetic acid content determined by serum metabolomics on day 0 and day 8 post-infection in mice (*n* = 3 to 4). c, Survival curves in groups treated with PBS, Abx, or Abx-treated mice pretreated with different short-chain fatty acids (SCFAs; 200 mM acetate [Ace], propionate [Pro] or butyrate [But]) prior to IAV PR8 infection (*n* = 8 to 10). d-f, Mice were treated with PBS, Abx, or SCFAs prior to IAV infection at 8 dpi. (d), Viral titers in the lung (*n* = 5 to 6). (e), Frequency of CD8^+^ T cells and influenza nuclear protein (NP)-positive CD8^+^ T cells in the lung (*n* = 3 to 4). (f), FACS analysis of IFN-γ production by CD8^+^ T cells in the lung (*n* = 3 to 4). g, Viral titers in the lung collected at 8 dpi from Abx- and SCFA-treated, PR8-infected WT or CD8^−/−^ mice (*n* = 3 to 5). h, Frequency of CD8^+^ T cells, influenza nuclear protein (NP)-positive CD8^+^ T cells and proportion of IFN-γ production by CD8^+^ T cells in the lung of Abx- and acetate-treated WT or GF mice at 8 dpi after IAV PR8 infection were detected (*n* = 3 to 6). i, Viral titers in the lung of Abx- and acetate-treated WT or GF mice at 8 dpi after IAV PR8 infection (*n* = 4). Plotted data represent the mean ± s.d.; unpaired two-tailed Student’s *t*-tests were used to compare means between treatment groups. *, *p* < 0.05; **, *p* < 0.01; ***, *p* < 0.001; ****, *p* < 0.0001; ns, not significant.

To test whether acetate plays a crucial role in protecting the host from IAV PR8 infection, groups of Abx-treated mice were given different SCFAs (acetate, propionate or butyrate) via oral gavage for 14 consecutive days (Figure S4f). Propionate was included in the protection study as an SCFA control and butyrate served as a positive control since it has previously been determined to mitigate mortality caused by IAV PR8 infection.^18^ As expected, oral administration of SCFAs significantly increased their concentration in the serum and feces of Abx-treated mice (Figure S4g,h). Acetate exerted an IAV PR8-protective effect equivalent to that of butyrate and markedly increased survival rate compared with untreated Abx control mice. There was also a trend for propionate treatment in reducing the lethality by IAV PR8 infection though the difference was not statistically significant (Figure 4(c)). We then harvested the lung tissues of different groups at 8 dpi for virus load detection and discovered significantly higher viral load in the lung of untreated or propionate-treated Abx mice compared with the acetate- or butyrate-treated mice, indicating that acetate was capable of mediating viral clearance in the pulmonary tissues during IAV PR8 infection (Figure 4(d)).

To evaluate the hypothesis that BC-derived acetate helps mice limit IAV PR8 infection by inducing CD8^+^ T cell responses, we tested the recruitment and activation of CD8^+^ T cells in the lung and spleen of SCFA-treated Abx mice at 8 dpi. Acetate and butyrate administration led to the significantly increased frequency of total and PR8-specific CD8^+^ T cells in the lung and spleen of Abx mice (Figure 4(e) and Figure S4i). Further, acetate and butyrate pretreatment caused a significant increase in frequency of CD69-expressing, IFN-γ- and granzyme B-secreting CD8^+^ T cells in the lung of Abx mice (Figure 4(f) and Figure S4j). Similar to the mucosal phenotype, activated CD8^+^ T cells expressing IFN-γ and granzyme B were augmented in the spleen of Abx mice pretreated with acetate and butyrate. In addition, compared with the Abx group, activated CD8^+^ T cells expressing CD69 were significantly increased in the spleen of Abx mice pretreated with acetate and also tended to increase after butyrate treatment, although the difference was not significant (Figure S4k). However, the percentage of CD4^+^ T cells did not show significant change in all groups in the spleen (Figure S4l).

To further corroborate the role of CD8^+^ T cell response in the acetate-induced antiviral state, Abx-treated WT or CD8^−/−^ mice were gavaged with SCFAs and then inoculated with IAV PR8 as mentioned above. No differences in viral titers or histopathology were seen in the lung of PR8-infected, Abx-treated CD8^−/−^ mice regardless of SCFA treatment, whereas their Abx-treated WT counterparts exhibited lower viral burden, ameliorated tissue damages as well as the fluorescence density of HA in lung tissue when pretreated with acetate or butyrate (Figure 4(g) and Figure S4m–o).

Subsequently, we determined whether the acetate-mediated CD8^+^ T cell activation observed in conventional WT mice with normal gut microbiota could be repeated in GF mice. After pretreating GF mice with acetate for 2 weeks, we inoculated with IAV PR8 and harvested the lung and spleen for CD8^+^ T cell detection. These experiments were conducted concurrently with the experiments shown in Figure 3(f–g); thus, the data from control GF mice are repeated in the figure for comparison. Indeed, acetate administration significantly augmented total and IAV PR8-specific CD8^+^ T cells in the lung and spleen of GF mice without affecting total CD4^+^ T cells in these tissues at 8 dpi (Figure 4(h) and Figure S4p,q). Moreover, there was higher expression level of CD69, IFN-γ and granzyme B in CD8^+^ T cells in the lung and spleen of acetate-treated GF mice compared with untreated GF mice at 8 dpi.

Additionally, a significantly higher level of IAV PR8 viral mRNA was detected in the lung of untreated WT (in the left) or GF mice (in the right) than in acetate-treated WT or GF mice (Figure 4(i)). Moreover, noncolonized GF mice exhibited more viral HA proteins as well as more severe tissue damages than GF mice treated with acetate (Figure S4r–t). In addition, Abx-treated CD8^−/−^ mice were gavaged with BC and BC-derived acetate and then inoculated with IAV PR8. On day 3 and day 5 post-infection, differences in viral titers or histopathology were observed in the lung (Figure S4u–w). Collectively, these data suggest that BC and BC derived acetates seem to activate innate immunity, which may also help protect against infection, while significantly enhancing adaptive immunity and regulating the function of CD8^+^T cells to clear viruses.

### Acetate enhances antiviral CD8^+^ T cell response by altering their metabolism in a GPR43-dependent manner

**Figure 5:**
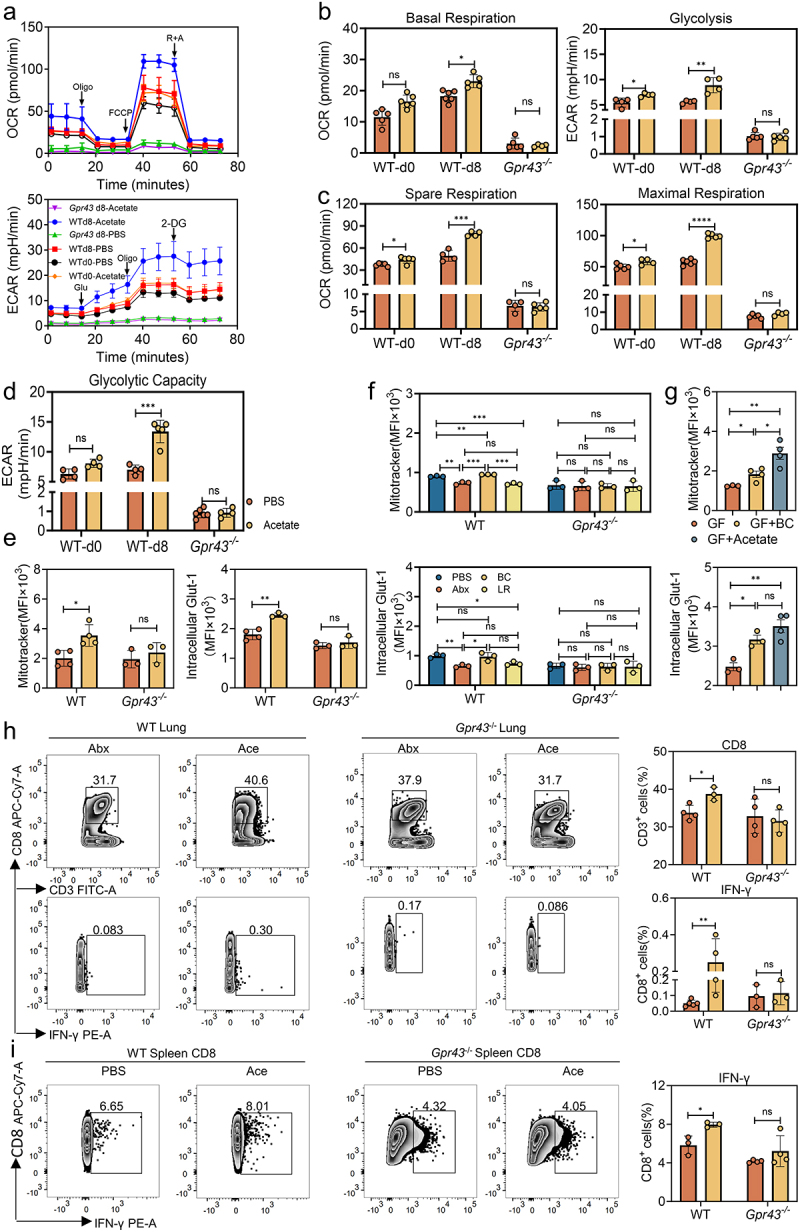
Acetate enhances adaptive immunity by altering CD8^+^ T cell metabolism in a GPR43-dependent manner.

SCFAs exert their functions via binding to GPR43 and GPR41,^21^ and it has been shown that GPR41 is required for butyrate-induced anti-IAV CD8^+^ T cell response and metabolism.^18^ To investigate whether the induction of antiviral CD8^+^ T cells mediated by BC-derived acetate was similarly linked to GPR43-dependent alteration of cellular metabolism, groups of Abx-treated WT or *Gpr43*^−/−^ mice were administered acetate and then inoculated with IAV PR8. At 8 dpi, splenic CD8^+^ T cells were isolated from both groups, and oxidative mitochondrial respiration and glycolytic metabolism were evaluated by measuring oxygen consumption rate (OCR) and extracellular acidification rate (ECAR) (Figure 5(a)). Compared with untreated Abx WT mice, CD8^+^ T cells from acetate-treated Abx WT mice had a significantly increased basal OCR and ECAR (Figure 5(b)). Furthermore, CD8^+^ T cells from acetate-treated WT mice displayed maximal respiration and spare respiratory capacity (SRC) superior to the those from untreated Abx WT mice (Figure 5(c)). *In vitro* stimulation also led to a significant increase in the glycolytic capacity of the CD8^+^ T cells from acetate-treated Abx WT mice, suggesting an enhanced capacity to rely on anaerobic glycolysis (Figure 5(d)). Similarly, significant differences in mitochondrial mass and intracellular expression of the glucose transporter Glut-1 were seen between acetate-treated and untreated Abx WT CD8^+^ T cells at 8 dpi (Figure 5(e)). On the contrary, oxidative mitochondrial respiration and glycolytic metabolism (Figure 5(a–d)) of splenic CD8^+^ T cells from acetate-treated and untreated Abx *Gpr43*^−/−^ mice were comparable at 8 dpi, as were the mitochondrial mass and intracellular Glut-1 expression (Figure 5(e)).

a-d, CD8^+^ T cells were isolated from the spleen of WT or *Gpr43*^*-/-*^ mice at 0 or 8 dpi, then stimulated or not with 5 mM acetate for 24 h. (a), Oxygen consumption rate (OCR) and extracellular acidification rate (ECAR) measurements were performed on CD8^+^ T cells. (b), Basal (OCR) and (ECAR) of CD8^+^ T cells (*n* = 4 to 6). (c), Spare respiratory capacity and maximal respiration (*n* = 4 to 6). (d), Glycolytic capacity of splenic CD8^+^ T cells (*n* = 4 to 6). e, Mitochondrial mass as measured by Mitotracker staining as well as intracellular Glut-1 expression of CD8^+^ T cells collected from the spleen of WT or *Gpr43*^*-/-*^ mice at 8 dpi, then stimulated or not with 5 mM acetate for 24 h (*n* = 3 to 4). f, Mitochondrial mass as measured by Mitotracker staining as well as intracellular Glut-1 expression on pulmonary CD8^+^ T cells from PBS-treated, Abx-treated, BC- or LR-colonized WT or *Gpr43*^*-/-*^ mice at 8 dpi after IAV PR8 infection (*n* = 3). g, Mitochondrial mass as measured by Mitotracker staining and intracellular Glut-1 expression on pulmonary CD8^+^ T cells with or without acetate-treated, BC-colonized GF mice at 8 dpi after IAV PR8 infection (*n* = 3 to 4). h, FACS analysis of CD8^+^ T cells and their effect-function indicators IFN-γ in the lung of Abx-treated WT or *Gpr43*^*-/-*^ mice with or without acetate at 8 dpi after IAV PR8 infection (*n* = 3 to 5). i, In vitro culture system, measurement of IFN-γ production in acetate-treated CD8^+^ T cells collected from PR8-infected WT or *Gpr43*^*-/-*^ mice at 8 dpi compared to the untreated control cells (*n* = 3 to 4). Plotted data represent the mean ± s.d.; unpaired two-tailed Student’s *t*-tests were used to compare means between treatment groups. *, *p* < 0.05; **, *p* < 0.01; ***, *p* < 0.001; ****, *p* < 0.0001; ns, not significant.

Consistent with the results observed in the acetate-treated Abx mice, BC colonization in Abx WT mice, but not *Gpr43*^−/−^ mice, caused significant augmentation of mitochondrial mass and intracellular expression of Glut-1 in pulmonary CD8^+^ T cells at 8 dpi, whereas LR colonization failed to do so in either mouse lines (Figure 5(f)). In addition, GF mice colonized with BC or administered acetate also exhibited higher levels of mitochondrial mass and intracellular Glut-1 in pulmonary CD8^+^ T cells at 8 dpi, reflecting an enhancement of cellular metabolism (Figure 5(g)).

To test whether the GPR43-dependent increase in metabolic function accounts for the acetate-driven activation of virus-specific CD8^+^ T cells, we detected the percentage of total and functionally activated CD8^+^ T cells of the lung or spleen harvested from acetate-treated Abx WT or *Gpr43*^−/−^ mice. As expected, there were no differences between the acetate-treated and control groups in *Gpr43*^−/−^ mice with respect to the total proportion of CD8^+^ T cells as well as their expression of CD69, IFN-γ and granzyme B in the lung (Figure 5(h) and Figure S5a) or spleen (Figure S5b) at 8 dpi. Further, the acetate-mediated antiviral effect was also abrogated in Abx *Gpr43*^−/−^ mice, as there was no difference in pulmonary histological lesions, pathological score, and mean fluorescence intensity of lung regardless of acetate treatment in these mice (Figure S5c and Figure S5d). As expected, there is no doubt that acetate-treated WT mice can reduce viral titers but not *Gpr43*^−/−^ mice (Figure S5e).

The GPR43-linked metabolic variation between acetate-treated and untreated Abx mice could be attributed to a direct effect of acetate on CD8^+^ T cells, as supplementation of acetate in splenic CD8^+^ T cells isolated from PR8-infected WT mice at 8 dpi significantly augmented mitochondrial mass and intracellular Glut-1 expression (Figure S5f) and concomitantly upregulated the expression of CD69, IFN-γ and granzyme B. However, this phenotype was impaired in splenic CD8^+^ T cells isolated from *Gpr43*^−/−^ mice (Figure 5(i) and Figure S5g). Collectively, these *in vivo* and *in vitro* results indicated that GPR43 is essential for induction of acetate-dependent metabolic changes and immune activation in virus-specific CD8^+^ T cells.

### Acetate restores ifn-γ production in T cells and changes in cellular metabolism

**Figure 6:**
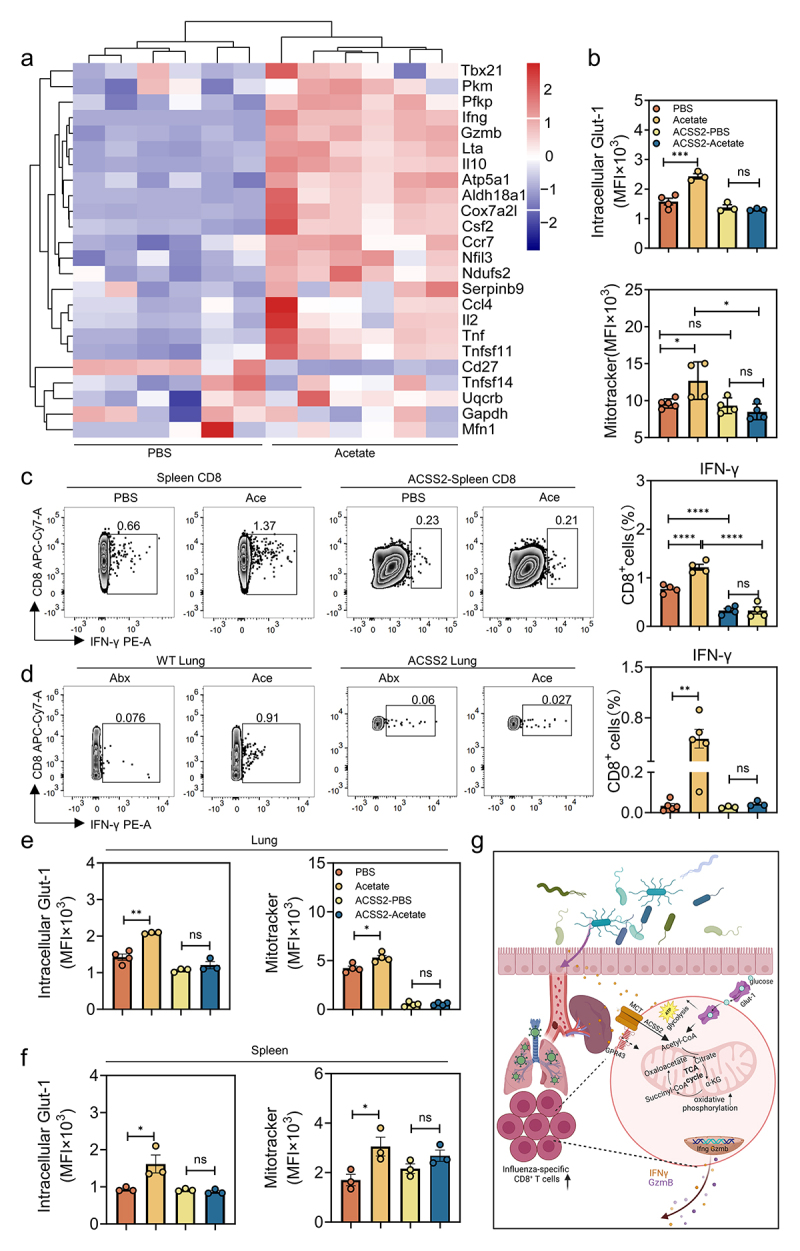
Acetate treatment enhances ifn-γ production in T cells and alters CD8^+^ T cell metabolism.

To define how acetate directly mediates metabolic reprogramming and antiviral immune response against IAV PR8, we performed RNA sequencing (RNA-seq) analysis on acetate-treated or untreated CD8^+^ T cells extracted from the spleen of PR8-infected WT mice at 8 dpi. We found that acetate supplementation substantially affected the transcriptional landscape of CD8^+^ T cells, with 154 differentially expressed genes (DEGs) compared with PBS-treated controls (Figure S6a). Gene ontology (GO) analysis revealed that the most significantly enriched pathways in acetate-treated vs untreated CD8^+^ T cells of infected WT mice were immune response-related pathways and glucose metabolism pathways (Figure S6b). Among those, we discovered genes encoding IFN-γ, granzymes, tumor necrosis factor (TNF), and the transcription factor T-bet (*Tbx21*), which controls IFN-γ expression (Figure 6(a)). Of note, acetate increased the expression of some of the transcripts related to oxidative phosphorylation (OXPHOS) and glycolysis including in CD8^+^ T cells of infected WT mice (Figure 6(a)), which was consistent with the boosted metabolism phenotype observed previously.

a, Transcriptome analysis was performed on acetate-treated CD8^+^ T cells collected from spleen of IAV PR8-infected WT mice at 8 dpi compared to the untreated control cells. The expression of 24 transcriptional genes involved in immune response, glycolysis and oxidative phosphoric acid were analyzed (*n* = 6). b, In vitro culture system. Proportion of mitochondrial mass and intracellular expression of Glut-1 in virus-specific CD8^+^ T cells collected from the spleen of PR8-infected WT mice at 8 dpi compared to the untreated control cells. CD8^+^ T cells were treated with 0.8 µM ACSS2 inhibitor for 24 h, followed by 24 h of incubation with acetate (*n* = 3 to 5). c, In vitro culture system. Proportion of IFN-γ in virus-specific CD8^+^ T cells was detected (*n* = 4). d, FACS analysis of IFN-γ expression of CD8^+^ T cells from the lung of Abx-treated with or without acetate mice at 8 dpi after lAV PR8 infection. WT mice were treated with or not ACSS2 inhibitor (*n* = 3 to 5). e-f, FACS analysis of intracellular Glut-1 and mitochondrial mass expression of CD8^+^ T cells from the lung (e) or spleen (f) of Abx-treated with or without acetate mice at 8 dpi after lAV PR8 infection. WT mice were treated with or not ACSS2 inhibitor (*n* = 3 to 4). g, Schematic illustration of the Blautia spp.-associated metabolite acetate how associated with functionally optimal virus-specific CD8^+^ T cell responses to influenza virus infection via GPR43-dependent metabolic reprogramming. Plotted data represent the mean ± s.d.; unpaired two-tailed Student’s *t*-tests were used to compare means between treatment groups. *, *p* < 0.05; **, *p* < 0.01; ***, *p* < 0.001; ****, *p* < 0.0001; ns, not significant.

A few studies point toward the conversion of microbiota-derived SCFAs into acetyl-CoA, which then fuels the OXPHOS and glycolysis pathways to affect the immune responses of CD8^+^ T cells.^18,22^ These processes would require direct entry of acetate into the CD8^+^ T cells through monocarboxylate transporters (MCTs)^23^ and the subsequent conversion to acetyl-CoA by ACSS2.^24^ Indeed, increased expression of MCT-1, MCT-4 and ACSS2 was observed in acetate-treated CD8^+^ T cells collected from IAV PR8-infected WT mice at 8 dpi compared with the untreated control cells (Figure S6c). Thus, acetate-treated virus-specific CD8^+^ T cells may upregulate MCTs to increase exogenous acetate uptake and convert the cytosolic acetate to acetyl-CoA by ACSS2, which fuels the OXPHOS and glycolysis pathways. To test this hypothesis, CD8^+^ T cells collected from IAV PR8-infected WT mice at 8 dpi were treated with an ACSS2 inhibitor, HY-104032, 24 h prior to acetate administration, stained for IFN-γ and granzyme B, and analyzed by flow cytometry. HY-104032 pretreatment efficiently decreased acetyl-CoA synthesis in the CD8^+^ T cells (Figure S6d). As expected, inhibition of ACSS2 by HY-104032 resulted in significantly lower mitochondrial mass and intracellular Glut-1 expression, with lower expression of IFN-γ and granzyme B in virus-specific CD8^+^ T cells compared with PBS-treated control CD8^+^ T cells. Importantly, acetate administration did not boost IFN-γ and granzyme B secretion in these CD8^+^ T cells in the presence of HY-104032 in vitro (Figure 6(b,c) and Figure S6e).

To further assess the effect of acetate on ACSS2-mediated metabolic alterations and immune activation in vivo, groups of WT mice were intraperitoneally injected with HY-104032 every other day after Abx treatment up to day 8 after IAV PR8 infection, either alone or in combination with acetate throughout the experiment (Figure S6f). The effect of ACSS2 inhibition was validated by detecting the level of acetyl-CoA in the serum of treated animals (Figure S6g). Consistent with the in vitro data, acetate administration failed to augment mitochondrial mass/intracellular Glut-1 or enhance production of IFN-γ/granzyme B in pulmonary (Figure 6(d–e) and Figure S6h) or splenic (Figure 6(f) and Figure S6i) CD8^+^ T cells collected from IAV PR8-infected WT mice in the presence of HY-104032 at 8 dpi. Furthermore, HY-104032 treated, IAV PR8-infected WT mice displayed clearly impaired viral clearance (Figure S6j) and worsened histopathologic lesions in the lung (Figure S6k), attributable to the effect on metabolism and activation in virus-specific CD8^+^ T cells resulting from the inhibition of acetyl-CoA synthesis. The pathological score and mean fluorescence intensity in lung tissue between acetate-treated and untreated PR8-infected WT mice were lost in the presence of HY-104032 at 8 dpi (Figure S6l). Together, these findings suggest that both BC and acetate exert its enhancement of cellular metabolism and induction of optimal virus-specific CD8^+^ T cell responses via promotion of acetyl-CoA synthesis.

## Discussion

Recently, we and others have demonstrated that microbiota-derived acetate can restrict both mucosal and systemic viral infection by priming IFN-I-mediated innate immune responses in local and peripheral mononuclear phagocytes.^15,19,25^ Using IAV PR8 as a model, we further focused on how the gut-lung axis crosstalk influences host antiviral adaptive immunity at extraintestinal mucosal sites, and how this response may impact infection outcomes. Here, we compared the intestinal microbiota of surviving mice vs those who succumbed to PR8 infection, identifying the commensal bacterium BC, whose production of acetate was shown to promote the host virus-specific CD8^+^ T cell response to limit local and systemic IAV infection by boosting GPR43-dependent cell metabolism (Figure 6(g)).

In our study, a significantly lower abundance of BC was demonstrated throughout the PR8 infection in those mice who eventually died (Figure 1(b)), implying a potential role for BC as a microbial biomarker in prediction of IAV infection outcomes in mice. Interestingly, we noted that the relative abundance of *Faecalibacterium* was also significantly higher in the survivors compared with those that died (Figure 1(b)), pointing toward a positive correlation between the intestinal concentration of this genus and the host resilience to IAV PR8 infection. Given the fact that one species, *Faecalibaculum rodentium*, is a robust SCFA-producer that has been reported to be capable of protecting against intestinal tumor growth,^26^ it is reasonable to speculate that it might also play a potential role in protecting mice from IAV PR8 infection. As such, we plan to perform functional studies in Abx and GF mouse models to test this hypothesis in the future.

Although our present study focused on BC-mediated antiviral adaptive immune responses, we did observe lower viral burden, ameliorated histopathologic lesions and enhanced levels of type I and type II IFN in the lung of PR8-colonized, BC-colonized Abx mice at 3 dpi (Figure 2), indicating that BC also controlled PR8 replication at the early phase of infection by promoting host innate immunity. It is a plausible explanation that BC may also exert this enhancing effect on innate immunity through acetate production, because our data are consistent with a recent study demonstrating that *Bifidobacterium pseudolongum*-derived acetate promotes oligomerization and signaling of MAVS through GPR43 activation and NLRP3 engagement in macrophages, leading to augmented IFN-I production against IAV.^15^ Nevertheless, the clearly enhanced protective immunity against IAV PR8 infection could not only be attributed to the altered IFN-I-mediated innate immunity, as adaptive immune responses are needed to clear the remaining viral loads in the lung during later phases of infection.

Metabolic profile screening of BC-colonized Abx mice revealed augmentation of serum SCFAs, especially acetate, both prior to and at day 8 post-IAV infection (Figure 4(a) and Figure S4a). This finding is perfectly consistent with the fact that nearly one-third of the acetate in the colon was produced by acetogenic bacterial communities such as *Blautia*.^27^ Administration of exogenous acetate reproduced the protective effect of BC colonization in both Abx and GF mice, enhancing viral clearance and alleviating the resulting histopathologic lesions by inducing virus-specific CD8^+^ T cells (Figure 4). BC produces acetate from acetyl-Pi by an acetate kinase encoded by the gene *ackA*.^28^ Because the entire field lacks an efficient, standardized, and i*n vitro* approach to genetically manipulate non-model intestinal bacteria, such as BC, one limitation of the current research is that we were unable to silence *ackA* to block acetate synthesis in BC and thus establish a direct correlation between BC and acetate, which would enable us to dissect the specific role of BC-derived acetate for inducing optimal CD8^+^ T cell response and limiting IAV infection in vivo.

Our data suggest that acetate enhances the effector functions of both mucosal and peripheral CD8^+^ T cells by altering their metabolism. Our in vitro and in vivo data showed that exogenous acetate administration increased both OXPHOS and glycolysis of virus-specific CD8^+^ T cells (Figure 5(a–d)). These results make perfect sense because during the differentiation from naïve to effector CD8^+^ T cells driven by viral infections, the cellular glucose metabolic form gets switched from OXPHOS to glycolysis to guarantee the generation of metabolic intermediates vital for cell growth and proliferation, resulting in a concomitant high glycolytic rate and OXPHOS activity at this particular phase.^29,30^ Furthermore, acetate promoted the intracellular expression of Glut-1 in the treated animals (Figure 5(e)), thereby providing a larger reservoir of the main glucose transporter in CD8^+^ T cells, which elevates glucose uptake to increase the functional capacity of these effector cells.

The current work demonstrated that administration of acetate induces metabolic alteration and immune stimulation by activating GPR43 in virus-specific CD8^+^ T cells (Figure 5). Although GPR41 and GPR43 are both SCFA receptors, acetate is more selective for GPR43, whereas butyrate is more active on GPR41.^31^ This preference may explain the differences between our investigation and the study conducted by Trompette et al., as they demonstrated that the metabolic and functional changes in CD8^+^ T cells induced by butyrate are partly dependent on GPR41,^18^ while our work indicates that the metabolic and functional changes induced by exposure of CD8^+^ T cells to acetate rely on GPR43 activation. The molecular link between cellular metabolism and GPR43 activation remains unclear, although it is possible that extracellular signal-regulated kinase (ERK) might be activated by GPR43 signaling, which has been shown to induce glucose import and glycolytic activities in T cells.^32^

In addition to its capacity as a GPR stimulator, our data suggest that acetate can also directly enter CD8^+^ T cells through MCTs and promote acetyl-CoA synthesis by enhancing ACSS2 (Figure 6). The augmented acetyl-CoA can then fuel the pathways of OXPHOS and glycolysis and activate the effector functions of CD8^+^ T cells. This finding is in line with one paper reporting that acetate enhances IFN-γ gene transcription and cytokine production, which rescued effector function in glucose-restricted tumor-infiltrating CD8^+^ T cells in an ACSS2-dependent manner.^33^ Similarly, Balmer et al. also showed that significantly increased serum acetate concentrations under the circumstances of bacterial systemic infection enhance glycolysis and cytosolic acetyl-CoA levels, which resulted in increased functional activity of CD8^+^ memory T cells.^22^

Our work builds on the limited existing literature by identifying an acetogenic commensal bacterium, BC, that plays a crucial role in protecting against IAV infection by inducing both mucosal and systemic optimal virus-specific CD8^+^ T cells. Additionally, our work highlights a fundamental role of microbiota-derived acetate in regulating effector functions of virus-specific CD8^+^ T cells by reprogramming their cellular metabolism in an ACSS2- and GPR43-dependent manner. Our observation that BC and its associated metabolite acetate can mitigate IAV PR8 infection in a mouse model may provide a basis for the development of microbiota-based approaches for the prevention and treatment of clinical IAV infections in humans.

## Materials and methods

### Viruses, bacteria and cell culture

A mouse-adapted, low-pathogenicity isolated of IAV H1N1 strain PR8 (abbreviated as PR8 hereafter) was used in this study. A virus stock was amplified in embryonated chicken eggs or MDCK cells. The IAV titer was determined with hemagglutination assays.

*B. coccoides* was purchased from ATCC (ATCC 29,236) and expanded anaerobically in modified chopped-meat medium (ATCC medium 1490; ELITE-MEDIA) at 37°C. *L. reuteri* was purchased from the China Center for Type Culture Collection (CCTCC, catalog no. AB 2,014,289) and cultured anaerobically in De Man-Rogosa-Sharpe medium (Oxoid) at 37°C.

MDCK cells were purchased from Shanghai Cell Bank of Chinese Academy of Sciences and cultured in Dulbecco’s modified Eagle medium (DMEM) supplemented with 1% penicillin/streptomycin (P/S), 10% fetal bovine serum (GIBCO, Invitrogen Corporation, Carlsbad, CA, USA), at 5% CO_2_ and 37°C. A549 cells were purchased from Shanghai Cell Bank of Chinese Academy of Sciences and cultured in F12 medium containing 10% fetal bovine serum (FBS, GIBCO) at 37°C and 5% CO_2_.

Mouse lung primary CD8^+^ T cells were obtained as follows. First, the lung tissue was cut into pieces 1–2 mm and then digested for 45 min in PBS containing 2% serum supplemented with 1 mg/mL collagenase IV and 0.2 mg/mL Dnase I (sigma) with gentle shaking at 5 min intervals. The digested lung tissue was then ground to obtain a single-cell suspension, which was cultured in RPMI-1640 medium containing 10% heated-inactivated FBS at 37°C and 5% CO_2_. Mouse spleen primary CD8^+^ T cells were prepared by grinding the spleen, and a single-cell suspension was obtained without digestion.

### Sample collection

WT B6 mice were infected with 10^6^ TCID50 of PR8 in 40 μL of PBS. Thereafter, the animal body weights and survival were monitored, and fecal samples were collected daily after the infection. Further, based on whether they succumbed to or survived from the infection, the mice in the 16S rRNA amplicon sequencing group (abbreviated as 16S group hereafter) were divided into the death group (the feces of dead mice collected on days 0, 3, 5, and 8 after infection, abbreviated as d0, d3, d5, d8 hereafter) and survival group (the feces of survival mice collected on days 0, 3, 5, and 8 after infection, abbreviated as s0, s3, s5, s8 hereafter), respectively.

### Animals and viral infections

Sex-matched, six-week-old C57BL/6 mice were purchased from the Model Animal Research Center from Nanjing University (Nanjing, China) and co-housed in a specific-pathogen-free (SPF) facility with temperature and humidity control (22 ± 2°C, 50 ± 10% humidity), with a standard 12:12 hour light/dark cycle. All animal experiments were conducted strictly in accordance with a protocol approved by the Institutional Animal Care and Use Committee of Jilin Agricultural University. The GF mouse experiment was performed at the Laboratory Animal Center, Huazhong Agricultural University. What’s more, mice were raised in a sterile environment with a free diet mode.

Rag1 knockout mice (Rag1^−/−^) mice were purchased from the Model Animal Research Center of Nanjing University (Nanjing, China). CD8^*-/-*^ and CD4^*-/-*^ mice were a kind gift from Dr Mingzhu Zheng of Southeast University, Nanjing, China. Free fatty acid receptor 2 knockout mice (referred to as *Gpr43*^−/−^) were originally obtained from Cyagen Biosciences Inc (Suzhou, china).

For all infection experiments in vivo, wild-type B6 mice were nasally infected with 10^5^ or 10^6^ TCID_50_ of IAV H1N1 strain PR8 in 40 μL of PBS. At the indicated time points, the lungs, spleen, blood and fecal samples were collected. In particular, lung tissue was dissected, weighed, homogenized, and titrated by qPCR. Related primers and probes are listed in the Key Resources table. After infection, clinical symptoms were monitored daily until death.

### Antibiotic treatment, fecal microbial transfer and bacterial colonization

As previously mentioned, mice were administered with an antibiotic cocktail composed of ampicillin, neomycin, metronidazole and vancomycin daily for 5 days via oral gavage.

Antibiotics were then added to the drinking water, and the animals were maintained with Abx- or PBS-containing water for the duration of the experiments.

For FMT experiments, on day 8 post-infection, fecal samples were collected from co-housed mice that succumbed to (DG) or survived (SG) the IAV PR8 infection. Simply, 200 mg of fecal pellets were homogenized in 1 mL PBS at 45 Hz for 1 min using sterile silica beads, filtered with a 100 μM filter, and centrifuged at 6,000 × g for 15 min. The collected samples were resuspended in PBS containing 10% (v/v) glycerol and frozen at −80°C. Mice were given Abx orally as described above and except for the Abx-only control group, all mice were gavaged with 150 μL filtered fecal homogenate.

For bacterial colonization experiments, mice were given 10^10^ CFU of *B. coccoides* or *L. reuteri* in 150 μL PBS on day 6 after Abx administration. Forty-eight hours after bacterial colonization, fecal samples were collected to test colonization efficiency.

### Protection studies with metabolites

In the SCFA protection experiment, 200 mM of acetate, propionate, or butyrate (Sigma) was added to drinking water for 2 weeks prior to infection and continued until the end of experiment. Protective experiments with acetate were validated in *Gpr43*^*-/-*^, CD8^−/−^, CD4^−/−^ knockout mice using the method described above. Besides, HY-104032 was used to verify the protective effect of acetate in mice. Most importantly, CD8^+^ T cells were isolated from the spleen of *Gpr43*^*-/-*^ or HY-104032-treated mice, and the protective effect of acetate was verified in vitro as well as related experimental operations as described in the previous results.

### Fecal bacteria quantification

Fecal bacterial DNA content was determined by qPCR (primers used are listed in the Key Resources list). The TIANamp fecal DNA Kit (TIANGEN) was used to extract fecal bacterial DNA. SYBR Green real-time fluorescent quantitative PCR Premix reagent (TOYOBO) was used for qPCR.

### Viral load quantification

The collected tissue samples were weighed and homogenized to obtain tissue RNA and titrated by qRT-PCR. Briefly, tissue samples were homogenized at 45 Hz for 1 min and centrifuged at 6000 × g for 5 min. Total RNA was extracted with TRIzol reagent (Invitrogen), and qRT-PCR was performed using a one-step qRT-PCR kit (Toyobo) on an ABI 7500 Fast instrument. Related primers and probes are listed in the Key Resources table.

### Cytokine expression analysis

Total RNA was extracted from tissue samples or cell cultures using TRIzol reagent (Invitrogen) according to the manufacturer’s instructions. Gene expression was determined by quantitative PCR with reverse transcription using the HiScript II One Step qRT – PCR SYBR Green Kit (Vazyme).

A mouse enzyme-linked immunosorbent assay (ELISA) kit (MultiSciences) was used to quantify IL-6 protein in serum samples, following the manufacturer’s instructions.

### Histopathology processing, scoring, and immunofluorescence assays

Paraffin sections were cleaned and fixed, then blocked in blocking buffer for 1 h. Recombinant IAV H1N1 HA/hemagglutinin antibody rabbit mAb (Sino Biological, Beijing, 400-fold dilution) was added to the blocking buffer and incubated at 4°C overnight. After washing, sections were incubated with FITC (BioLegend, United States) coupled with secondary antibody at 1:400 dilution for 1 h at room temperature, then washed 3 times with immunostaining washing solution (Beyotime, Shanghai) and stained at room temperature with 4, 6-diamino-2-phenylindole (DAPI) for 5 min. Finally, the immunostaining washing solution was used to wash three times, and images were observed and acquired using a fluorescence microscope.

### Flow cytometry

Spleen, lung and BALF were harvested to analyze surface antigen levels on different cell subsets following blockade of Fcg receptors with anti-CD16/32. Fluorescently conjugated antibodies targeting NK1.1^+^, Mφ, CD19, CD3, CD4, CD8, CD69, IFN-γ, granzyme B, Glut-1, mitochondrial mass, CD11c and CD86 were used. Dendritic cells (DC) were identified as, CD11c^+^ and CD86^+^. NK cells were identified as NK1.1^+^. Macrophages were identified as F4/80^+^ and CD11b^+^. CD69^+^ cells, CD3^+^ cells, CD4^+^ T cells, CD8^+^ T cells, and B^+^ cells were identified as CD69^+^, CD3^+^, CD3^+^CD4^+^, CD3^+^CD8^+^, and CD19^+^, respectively. IFN-γ, granzyme B, Glut-1 and mitochondrial mass were identified as CD3^+^CD8^+^IFN-γ^+^, CD3^+^CD8^+^granzyme B^+^, CD3^+^CD8^+^Glut-1^+^ and CD3^+^CD8^+^ mitochondrial mass^+^.

### CD8^+^ T cells adoptive transfer

Splenic CD8^+^ T cells from 8-week-old wild-type female mice were obtained by negative selection using magnetic beads, and 2 × 10^6^ CD8^+^ T cells were intravenously transferred to CD8^−/−^ or Rag^−/−^ receptor mice. Cell purity was measured by flow cytometry before injection into the recipient mice. The day after adoptive transfer, CD8^−/−^ or Rag^−/−^ mice were intranasally infected with IAV PR8, then sacrificed at 8 dpi, and the frequency and effector function of influenza-specific CD8^+^ T cells present in the lungs were assessed by flow cytometry.

### In vitro CD8^+^ T cell assays

CD8^+^ T cells were isolated from the spleen of female mice aged 8–12 weeks, placed in 5 μg/mL anti-CD3- and 2 μg/mL anti-CD28-coated cell plates, and incubated at 37°C and 5% CO_2_ for 48 h. Then, the cells were treated with or without 5 µM sodium acetate for 24 h, and the metabolic status and activation of CD8^+^ T cells in the presence or absence of sodium acetate were compared by flow cytometry.

Purified CD8^+^ T cells were stimulated and activated according to the above methods, and their metabolic phenotypes were analyzed. OCR (pmol/min) and ECAR (mpH/min) were measured using the Seahorse XF-96 Metabolic Extracellular flux Analyzer (Agilent) by two different experimental functions, the mitochondrial stress test and the glycolytic stress test.

### DNA extraction, 16S rRNA amplicon sequencing and data analyses

Fecal samples (~200 mg) were fully resuspended in ASL buffer (Qiagen) and homogenized for 2 min. Total fecal DNA was obtained from supernatant using the QIAamp Fecal DNA Mini Kit (Qiagen), and its concentration and purity were measured using Qubit (Thermo Fisher Scientific). Fecal DNA was amplified by PCR targeting variable regions 3 and 4 (V3–V4) of the 16S rRNA gene (forward primer, ACTCCTACGGGAGGCAGCA; reverse primer, GGACTACHVGGGTWTCTAAT) using Phusion High-Fidelity PCR Master Mix (New England Biolabs). Multiple sequencing of amplicons with sample-specific barcodes was performed using the Illumina NovaSeq platform. Paired-end reads were merged into long sequences with FLASH v.1.2.7, an accurate analysis and fast tool designed to merge paired-end reads when there are overlaps between read 1 and read 2 (ref. 47). The merged sequences were then analyzed using the QIIME v.1.9.1 software package.

### GC-MS/MS determination of SCFA concentrations

Twenty-milligram fecal sample, bacterial culture supernatant or quantities of serum were placed in a 2 mL EP tube with a small steel pellet and 1 mL of phosphoric acid (0.5% v/v) solution. The samples were homogenized, vortexed for 10 min and then 5 min, centrifuged at 10,000 × g for 10 min at 4°C, and 100 μL of supernatant was obtained. About 0.5 mL of MTBE (including internal standard) solution was added to the supernatant, vortexed for 3 min, sonicated for 5 min, and centrifuged at 10,000 × g at 4°C for 10 min, and the supernatant was collected for GC-MS/MS analysis.

For the SCFA analysis by GC-MS/MS, an Agilent 7890B gas chromatogram was used in conjunction with a 7000D mass spectrometry with a DB-FFAP column (30 m length × 0.25 mm i.d. ×0.25 μm film thickness, J&W Scientific, USA). The carrier gas type was helium, with a flow rate of 1.2 mL/min. Injection was done in the split mode with an injection volume of 2 μL. The oven temperature was maintained at 90°C for 1 min, increasing to 100°C at 25 °C/min, then increasing to 150°C at 20 °C/min for 36 s, and then to 200°C at 25 °C/min for 0.5 min, and operated for 3 min. All samples were analyzed by multi-reaction monitoring mode. The injector inlet and transfer line temperatures were 200°C and 230°C, respectively. SCFA content was detected by MetWare (http://www.metware.cn/) based on the Agilent 7890B-7000D GC-MS/MS platform.

### Transcriptomics analysis

CD8^+^ T cells were extracted from the spleens of Abx-treated mice at 8 dpi. The total RNA was extracted with TRIzol reagent (Invitrogen) after treating CD8^+^ T cells in vitro for 24 hr with or without 1 mM acetate. Samples were evaluated in the Agilent 4200 system (Agilent Technologies), Qubit 3.0 (Thermo Fisher Scientific), and Nanodrop One (Thermo Fisher) Scientific). RNA-seq libraries were generated and sequenced by Guangdong Meijian Biotechnology Co., LTD. All three samples tested were constructed in separate libraries, sequenced and analyzed as recommended by the manufacturer; full messenger RNA-SEQ libraries were generated using the NEB Next Ultra Nondirectional RNA library Prep Kit for Illumina (New England Biolabs).

### Statistics and reproducibility

Statistical analyses were performed with Prism GraphPad software. Error bars represent standard errors of the means in all figures and *p* values were determined by two-tailed Student’s *t*-test or analysis of variance. R^2^ was estimated for the correlation analysis of two continuous variables. A log-rank test was used for survival curves. A two-sided *p* value <0.05 was considered statistically significant.

All experiments were repeated, with the number of replicates stated in the figure legends.

## Supplementary Material

Supplemental Material

## Data Availability

16S rRNA sequence data are available in the Sequence Read Archive (SRA) under BioProject accession PRJNA1111230 and PRJNA1111316. RNA-seq data are available in the SRA under BioProject accession PRJNA1014239. All other data supporting the conclusions of this study are available in the paper and supplemental materials.
