# Supplementary material for: Microbiota-derived acetate is associated with functionally optimal virus-specific CD8+ T cell responses to influenza virus infection via GPR43-dependent metabolic reprogramming
Source: Gut Microbes. 2024 Oct 10;16(1):2401649. doi: 10.1080/19490976.2024.2401649 (PMC11469431; doi:10.1080/19490976.2024.2401649)
Supplement: Supplemental Material [file KGMI_A_2401649_SM8967.zip › Supplementary_files__41_ (1)/KGMI_A_2401649/Supplementary figure.docx]

**Extended Data Fig. 1 Commensal microbiota from surviving mice increases the resistance of WT mice to IAV PR8 infection**

**a**, Schematic representation of animal challenge experiments in mice treated with Abx, FMT or bacterial colonization followed by IAV PR8 infection. **b-c**, 16S rRNA gene V3–V4 regions of the feces of Abx-SG and Abx-DG mice at 0 dpi **(b)**, The PCA of SG and DG groups. **(c)**, a detailed genus-level simper analysis of different species. **d**, Relative abundance of bacterial genera in the surviving mice (s0, s3, s5, s8) and dying mice (d0, d3, d5, d8) (n=3 to 4). **e**, qRT-PCR of bacterial genomic copies of *Blautia coccoides* in feces from PBS-treated or Abx-treated mice colonized by who succumbed to (Abx+DG) or survived IAV PR8 infection (Abx+SG) (n=7). **f-g,** qRT-PCR of bacterial genomic copies in feces from PBS-treated or Abx-treated mice after colonization by *B. coccoides* **(f)** or *L. reuteri* **(g)** (n=4). Plotted data represent the mean ± s.d.; unpaired two-tailed Student’s *t*-tests were used to compare means between treatment groups. *, p< 0.05; **, p< 0.01; ***, p< 0.001; ****, p< 0.0001; ns, not significant.

**Extended Data Fig. 2 Innate cellular immune responses were greatly diminished in Abx-treated mice following IAV PR8 infection**

**a**, Flow cytometer has these two scattered light channels, FSC-H and SSC-A. Flow cytometry gating strategy of splenic natural killer cells (NK), mononuclear phagocytes (Macrophage) and dendritic cells (DC) from IAV PR8-infected mice at 3 dpi. Flow cytometry gating strategy of splenic CD3^+^ (FITC), CD4^+^ (V450), CD8^+^ (APC-Cy7), CD69^+^ (APC), IFN-γ^+^ (PE), granzyme B^+^ (PE-Cy7) and B (APC) cells from IAV PR8-infected mice at 8 dpi. Flow cytometry gating strategy of splenic Glut-1^+^ (FITC) and Mitotrack^+^ cells (FITC) from IAV PR8-infected mice at 8 dpi. Gating strategy for flow cytometry of CD4^+^ and CD8^+^ T cells from CD4^-/-^ and CD8^-/-^ mouse blood. **b-c**, Frequency of a panel of adaptive immune cells, including CD4^+^, CD8^+^ (b) and B cells (c) from the lung or spleen of PBS-treated, Abx-treated, BC- or LR-colonized Abx mice at 3 dpi (n=3 to 5). **d**, Relative *Il6* expression (above) in the lung (n=4 to 8). The expression of IL-6 protein (below) in serum was detected by ELISA (n=4 to 5). Plotted data represent the mean ± s.d.; unpaired two-tailed Student’s *t*-tests were used to compare means between treatment groups. *, *p*< 0.05; **, *p*< 0.01; ***, *p*< 0.001; ****, *p*< 0.0001; ns, not significant.

**Extended Data Fig. 3** ***B. coccoides* colonization boosted antiviral CD8^+^ T cell responses**

**a-d**, PBS-treated, Abx-treated, BC- or LR-colonized Abx mice at 8 dpi after IAV PR8 infection. **(a)**, Frequency of CD4^+^ and CD8^+^ T cells in the lung or spleen (n=4 to 7). (**b)**, Frequency of influenza nuclear protein (NP)-positive CD8+ T cells in the spleen (n=5 to 6). **(c)**, Frequency of CD4^+^ and CD8^+^ T cells in the BALF (n=4 to 6). **(d)**, FACS analysis of CD69, granzyme B or IFN-γ production by CD8^+^ T cells in the spleen, lung and BALF (n=4 to 9). **e**, Viral titers in the lung of PBS-treated, Abx-treated, BC- or LR-colonized Abx mice at 8 dpi after IAV PR8 infection (n=4 to 6). **f**, Frequency of CD4^+^ T cells and CD8^+^ T cells in the spleen from PBS-treated, Abx-treated, BC- or LR-colonized Abx mice at 12 dpi after IAV PR8 infection (n=4 to 5). **g**, qRT-PCR of fecal bacterial genome copies in PBS-treated mice or Abx-treated WT mice with or without BC colonization, and GF mice with or without BC colonization (n= 3 to 4). **h-i**, Abx-treated or BC-colonized, PR8-infected WT or GF mice at 8 dpi after IAV PR8 infection**. (h)**, the splenic frequency of CD8^+^ T cells and NP-positive CD8^+^ T cells. Frequency of CD4^+^ T cells in the lung and spleen (n= 3 to 7). **(i)**, FACS analysis of CD69, granzyme B or IFN-γ production by CD8^+^ T cells in the spleen and lung (n=3 to 5). **j-k,** Lung collected at 8 dpi following IAV PR8 infection from PBS- or Abx-treated WT or GF mice, with or without bacterial colonization. **(j)**, Left, pulmonary histology during IAV PR8 infection (magnification, 20x); scale bar, 50 μm. Right, lung immunofluorescence analysis at 8 dpi of hemagglutinin (red) and nuclei (blue). **(k)**, Blinded sections were assessed to determine the level of pathological severity. In order to evaluate the overall histological changes, lung tissue sections were scored according to the criteria specifed in the panel. The scoring system used was as follows: 0, no pathological change; 1, affected area (≤20%); 2, affected area (≤50%, >20%); 3, affected area (>50%, ≤80%); 4, affected area (>80%). **l,** Quantitative analysis of the fluorescence intensity of HA in lung, showing the mean fluorescence intensity. **m, n**, Weight loss in PBS-treated, Abx-treated, or BC-colonized WT mice (m), and noncolonized or BC-colonized CD4^-/-^ and CD8^-/-^ mice (n) after IAV PR8 infection (n=3). **o**, Proportion of CD8^+^ T cells in the spleen of WT mice, CD8^+^ T cells after in vitro sorting and purification, and Rag1^-/-^ mice with adoptive or non-adoptive transfer of mice (n=3). **p**, Proportion of NP-specific CD8^+^ T cells transferred to Rag^-/-^ mice with adoptive transfers of Abx or BC-derived CD8^+^ T cells after IAV PR8 infection in the spleen of WT mice (n=3). **q**, Mouse weight loss in Rag^-/-^ mice with or without adoptive transfers of Abx, BC, or PBS-derived CD8^+^ T cells after IAV PR8 infection (n=6 to 8). Plotted data represent the mean ± s.d.; unpaired two-tailed Student’s *t*-tests were used to compare means between treatment groups. *, *p*< 0.05; **, *p*< 0.01; ***, *p*< 0.001; ****, *p*< 0.0001; ns, not significant.

**Extended Data Fig. 4 The *B. coccoides* metabolite acetate rescued the generation of IAV-specific CD8^+^ T cells**

**a**, Heatmaps show differences untargeted metabolomics analysis of PBS-treated, Abx-treated, BC- or LR-colonized Abx mice at 0 dpi after IAV PR8 infection (n=3 to 4). **b**, Principal component analysis (PCA) of four groups: PBS-treated, Abx-treated, BC- or LR-colonized Abx mice at 8 dpi after IAV PR8 infection (n=3 to 5).**c**, KEGG analysis of differentially regulated metabolites between the PBS group and the Abx group at 8 dpi after IAV PR8 infection. Two-sided p values were examined by Student’s *t*-test (n=3 to 4). **d,** Metabolome analysis of BC culture supernatant targeting short-chain fatty acids (n= 4 to 6). **e，**Determination of acetate content from mouse serum of Abx-SG or Abx-DG (n= 4 to 5). **f**, Schematic of protective validation experiments with SCFAs which Abx-treated mice were given short-chain fatty acids (SCFAs; acetate, propionate or butyrate) via oral gavage for 14 consecutive days. **g, h,** SCFA concentrations in **(g)** serum (n=5 to 6) and **(h)** feces (n=4) after 2 weeks of SCFA treatment in uninfected WT mice. **i-l**, Lung and spleen tissues were harvested from PBS mice, untreated, acetate-, butyrate-, propionate-treated Abx WT mice at 8 dpi following IAV PR8 infection. **(i)**, the splenic frequency of CD8^+^ T cells and NP-positive CD8^+^ T cells (n=4 to 7). **(j, k)**, FACS analysis of CD69, granzyme B or IFN-γexpression of CD8^+^ T cells from the lung **(j)** or spleen **(k)** (n=3 to 8). **(l)**, Frequency of CD4^+^ T cells in the spleen (n= 3 to 4). **m-o**, Lung tissues were harvested from PBS mice, untreated, acetate-, butyrate-, propionate-treated Abx CD8^-/-^ mice or Abx WT mice at 8 dpi following IAV PR8 infection. **(m)**, Left, pulmonary histology during IAV infection (magnification, 20x); scale bar, 50 μm. Right, lung immunofluorescence analysis at 8 dpi of hemagglutinin (red) and nuclei (blue). **(n)** Quantitative analysis of the fluorescence intensity of HA in lung, showing the mean fluorescence intensity. **(o)**, Blinded sections were assessed to determine the level of pathological severity. In order to evaluate the overall histological changes, lung tissue sections were scored according to the criteria specifed in the panel. The scoring system used was as follows: 0, no pathological change; 1, affected area (≤20%); 2, affected area (≤50%, >20%); 3, affected area (>50%, ≤80%); 4, affected area (>80%). **p-q,** Lung and spleen tissues were harvested from Abx- and acetate-treated WT or GF mice at 8 dpi after IAV PR8 infection. **(p)**, FACS analysis of CD69 and granzyme B production in CD8+ T cells and NP-positive CD8+ T cells in the lung (n=3 to 6). **(q)**, FACS analysis of CD8^+^ T cells and their effect-function indicators CD69, IFN-γ and granzyme B were performed on spleen tissue (n=3 to 5). The frequency of CD4^+^ T cells in the spleen and lung (n=3 to 4). **r**, Left, pulmonary histology during IAV infection (magnification, 20x); scale bar, 50 μm. Right, lung immunofluorescence analysis at 8 dpi of hemagglutinin (red) and nuclei (blue) in GF mice treated with Abx with or without acetate. **s,** Blinded sections were assessed to determine the level of pathological severity. In order to evaluate the overall histological changes, lung tissue sections were scored according to the criteria specifed in the panel. The scoring system used was as follows: 0, no pathological change; 1, affected area (≤20%); 2, affected area (≤50%, >20%); 3, affected area (>50%, ≤80%); 4, affected area (>80%). **t,** Quantitative analysis of the fluorescence intensity of HA in lung, showing the mean fluorescence intensity. **u-w,** Lung tissues were harvested from Abx-, BC- and acetate-treated CD8^-/-^ mice at 3 or 5 dpi after IAV PR8 infection. (u), pulmonary histology during IAV infection (magnification, 20x); scale bar, 50 μm. (v), Blinded sections were assessed to determine the level of pathological severity. In order to evaluate the overall histological changes, lung tissue sections were scored according to the criteria specifed in the panel. The scoring system used was as follows: 0, no pathological change; 1, affected area (≤20%); 2, affected area (≤50%, >20%); 3, affected area (>50%, ≤80%); 4, affected area (>80%). (w), Viral titers in lung collected from differently treated mice at 3 or 5 dpi following IAV PR8 infection (n=4). Plotted data represent the mean ± s.d.; unpaired two-tailed Student’s *t*-tests were used to compare means between treatment groups. *, *p*< 0.05; **, *p*< 0.01; ***, *p*< 0.001; ****, *p*< 0.0001; ns, not significant.

**Extended Data Fig. 5 Acetate enhances adaptive immunity by altering CD8^+^ T cell metabolism in a GPR43-dependent manner**

**a**, **b**, Lung and spleen tissues were harvested from Abx-treated with or without acetate, PR8-infected WT or *Gpr43^-/-^* mice at 8 dpi after IAV PR8 infection. **(a)**, FACS analysis of CD69 and granzyme B production in CD8^+^ T cells in the lung (n=3 to 5). **(b)**, FACS analysis of CD8^+^ T cells and CD69, granzyme B and IFN-γproduction by CD8^+^ T cells in the spleen (n=3 to 4). **c,** Left, pulmonary histology during IAV infection (magnification, 20x); scale bar, 50 μm. Right, lung immunofluorescence analysis at 8 dpi of hemagglutinin (red) and nuclei (blue) in *Gpr43^-/-^* mice treated with Abx with or without acetate. **d,** Left, blinded sections were assessed to determine the level of pathological severity. In order to evaluate the overall histological changes, lung tissue sections were scored according to the criteria specifed in the panel. The scoring system used was as follows: 0, no pathological change; 1, affected area (≤20%); 2, affected area (≤50%, >20%); 3, affected area (>50%, ≤80%); 4, affected area (>80%). Right, quantitative analysis of the fluorescence intensity of HA in lung. **e**, Viral titers in the lung of Abx- and acetate-treated WT or *Gpr43^-/-^* mice at 8 dpi after IAV PR8 infection (n=4). **f**, In vitro culture system, measurement of mitochondrial mass and intracellular Glut-1 expression of acetate-treated CD8+ T cells collected from IAV PR8-infected WT or Gpr43-/- mice at 8 dpi compared with untreated control cells (n=3 to 5). **g**, Measurement of CD69 and granzyme B production in acetate-treated CD8^+^ T cells collected from PR8-infected WT or *Gpr43^-/-^* mice at 8 dpi compared to the untreated control cells (n=3 to 4). Plotted data represent the mean ± s.d.; unpaired two-tailed Student’s *t*-tests were used to compare means between treatment groups. *, *p*< 0.05; **, *p*< 0.01; ***, *p*< 0.001; ****, *p*< 0.0001; ns, not significant.

**Extended Data Fig. 6 Acetate treatment enhances IFN-γ production in T cells and alters CD8^+^ T cell metabolism**

**a**, In vitro culture system. Volcano plot of differentially regulated metabolites from CD8^+^ T cells between the groups treated with PBS or acetate (n=6). **b**, GO analysis comparing enriched signaling pathways between the PBS group and the acetate group (n=6). **c**, Relative MCT-1, MCT-4, and ACCS2 expression in acetate-treated CD8^+^ T cells collected from PR8-infected WT mice at 8 dpi compared to untreated control cells (n=4 to 6). **d**, ACSS2 enzyme activity in supernatant of ACSS2 inhibitor and acetate-treated CD8+ T cells collected from PR8-infected WT mice at 8 dpi compared to acetate-only treated CD8+ T cells (n=3). **e**, In vitro culture system. FACS analysis of granzyme B expression of CD8^+^ T cells collected from the spleen of PR8-infected WT mice at 8 dpi compared to the untreated control cells. CD8^+^ T cells were treated with 0.8 µM ACSS2 inhibitor for 24 h, followed by 24 h of incubation with acetate (n=4). **f**, Schematic representation of in vivo ACSS2 inhibitor experiment of acetate. **g**, Detection of ACSS2 enzyme activity in serum collected from acetate-treated WT mice at 8 dpi with or without ACSS2 inhibitor treatment (n=3).**h, i,** FACS analysis of granzyme B or IFN-γ expression of CD8^+^ T cells from **(h)** the lung and **(i)** the spleen of Abx-treated with or without acetate mice at 8 dpi after PR8 infection. WT mice were treated with or not ACSS2 inhibitor (n=3 to 6). **j**, Viral titers in the lung of Abx- and acetate-treated or not, PR8-infected WT at 8 dpi after IAV PR8 infection. WT mice were treated with or not ACSS2 inhibitor (n=3 to 4). **k**, Left, pulmonary histology during IAV infection (magnification, 20x); scale bar, 50 μm. Right, lung immunofluorescence analysis at 8 dpi of hemagglutinin (red) and nuclei (blue) in mice treated with Abx with or without acetate and then ACSS2 inhibitor. **l**, Left, blinded sections were assessed to determine the level of pathological severity. In order to evaluate the overall histological changes, lung tissue sections were scored according to the criteria specifed in the panel. The scoring system used was as follows: 0, no pathological change; 1, affected area (≤20%); 2, affected area (≤50%, >20%); 3, affected area (>50%, ≤80%); 4, affected area (>80%). Right, quantitative analysis of the fluorescence intensity of HA in lung**.** Plotted data represent the mean ± s.d.; unpaired two-tailed Student’s *t*-tests were used to compare means between treatment groups. *, *p*< 0.05; **, *p*< 0.01; ***, *p*< 0.001; ****, *p*< 0.0001; ns, not significant.
