# Supplementary material for: Microbiota-derived acetate is associated with functionally optimal virus-specific CD8+ T cell responses to influenza virus infection via GPR43-dependent metabolic reprogramming
Source: Gut Microbes. 2024 Oct 10;16(1):2401649. doi: 10.1080/19490976.2024.2401649 (PMC11469431; doi:10.1080/19490976.2024.2401649)
Supplement: Supplemental Material [file KGMI_A_2401649_SM8967.zip › Supplementary_files__41_ (1)/KGMI_A_2401649/Table of Key Resources.docx]

Supplementary Table. List of antibody, agonists, chemicals, critical commercial assays and oligonucleotides primers information.

| **REAGENT or RESOURCE** | **SOURCE** | **IDENTIFIER** |
| --- | --- | --- |
| **Recombinant Proteins** |  |  |
| Recombinant Murine IL2 | peprotech | 212-12-5 |
| Ultra-LEAF™ Purified anti-mouse CD3ε Antibody | biolegend | 100340 |
| Ultra-LEAF™ Purified anti-mouse CD28 Antibody | biolegend | 102116 |
| Influenza NP-peptide | ZYBio | NP-pepetide |
| **Antibodies** |  |  |
| HA/hemagglutinin antibody rabbit mAb | Sino Biolo | 11684-R107 |
| MitoTracker^TM^ Green FM | eBioscience™ | M7514 |
| Anti-Glucose Transporter GLUT1 | abcam | ab115730 |
| Goat Anti-Rabbit IgG | abcam | ab150077 |
| Anti-Histone H3 (acetyl K27) | abcam | ab117178 |
| Anti-Histone H3 (acetyl K4+K9+K14+K18+K23+K27) | abcam | ab300641 |
| Flu.NP_366_ [ASNENMETM,H-2D(b)] | HELIXGEN | HG08T7030 |
| Fixable Viability Stain 570 | BD Biosciences | 564995 |
| NK1.1 Monoclonal Antibody | eBioscience™ | 11-5941-82 |
| PE/Cyanine5 anti-mouse F4/80 Antibody | Biolegend | 103113 |
| Brilliant Violet 421™ anti-mouse/human CD11b Antibody | Biolegend | 101235 |
| Alexa Fluor® 488 anti-mouse CD86 Antibody | Biolegend | 105018 |
| APC anti-mouse CD11c Antibody | Biolegend | 107611 |
| Granzyme B Monoclonal Antibody | eBioscience™ | 25-8898-82 |
| IFN gamma Monoclonal Antibody | eBioscience™ | 12-7311-82 |
| APC anti-mouse CD69 Antibody | eBioscience™ | 17-0691-82 |
| APC anti-mouse CD19 Antibody | Biolegend | 152409 |
| CD16/CD32 Monoclonal Antibody (93) | eBioscience™ | 14-0161-85 |
| CD4 Monoclonal Antibody (GK1.5), eFluor™ 450 | eBioscience™ | 48-0041-82 |
| CD8 alpha Monoclonal Antibody (53-6.7), APC-Cyanine7 | eBioscience™ | A15386 |
| CD3 Monoclonal Antibody | eBioscience™ | 17-0038-42 |
| CD3 Monoclonal Antibody | eBioscience™ | 11-0032-82 |
| **Critical Commercial Assays** |  |  |
| **Mouse CD8 T cell Isolation Kit** | **Stemcell** |  |
| **Acetyl-CoA synthetase 2** | **mlbio** | **ml516285V** |
| **Mouse IL-6 ELISA Kit** |  |  |
| **Chemicals** |  |  |
| Collagenase D | Roche | 11088866001 |
| Dnase I | Roche | 10104159001 |
| Cell Stimulation Cocktail | eBioscience™ | 00-4975-93 |
| Sodium acetate | Sigma | S2889 |
| Sodium propionate | Sigma | p1880 |
| Sodium butyrate | Sigma | 303410 |
| HEPES | MedChemExpress | HY-D0857 |
| Gibco™ MEM | Gibco | 11140050 |
| β-mercaptoethanol | eBioscience™ | 21985023 |
| Sodium pyruvate solution | Gibco | 11360070 |
| Dimethyl sulfoxide | Sigma | D2650 |
| Corn oil | MedChemExpress | HY-Y1888 |
| **Oligonucleotides Primers** | **Sense primer** | **Antisense primer** |
| PR8 | GACCRATCCTGTCACCTCTGAC | GGGCATTYTGGACAAAKCGTCTACG |
| PR8 Probe | TGCAGTCCTCGCTCACTGGGACG |  |
| *Ifnb* | CGGACTTCAAGATCCCTATGGA | TGGCAAAGGCAGTGTAACTCTTC |
| *IL-6* | AGGATACCACTCCCAACAGA | ACTCCAGGTAGCTATGGTACTC |
| *IL-1b* | AAGCCTCGTGCTGTCGGACC | TGAGGCCCAAGGCCACAGGT |
| *TNF-α* | CATCTTCTCAAAATTCGAGTGACAA | CCAGCTGCTCCTCCACTTG |
| *β-actin* | ACTCGTCATACTCCTGCT | GAAACTACCTTCAACTCC |
| *Gpr43* | ACAGTGGAGGGGACCAAGAT | GGGGACTCTCTACTCGGTGA |
| *B.coccoides* | GGAGCGTAGACGGAAGAG | CCCGACACCTAGTATTCATC |
| *L.reuteri* | TTGGAAATGTTCCACAAGAC | TTGTGAGTTTGGATTGAACC |
| *C.butyrate* | GTGCCGCCGCTAACGCATTAAGTAT | CCATGCACCACCTGTCTTCCTGCC |
| *16s* | GGGCAAGCGTTATCCG | TCGCCACTGGTGTTCCT |
| *ACSS2* | GTGAAAGGATCTTGGATTCCAGT | CAGATGTTTGACCACAATGCAG |
