# Supplementary figures and images for: Microbiota-derived acetate is associated with functionally optimal virus-specific CD8+ T cell responses to influenza virus infection via GPR43-dependent metabolic reprogramming
Source: Gut Microbes. 2024 Oct 10;16(1):2401649. doi: 10.1080/19490976.2024.2401649 (PMC11469431; doi:10.1080/19490976.2024.2401649)

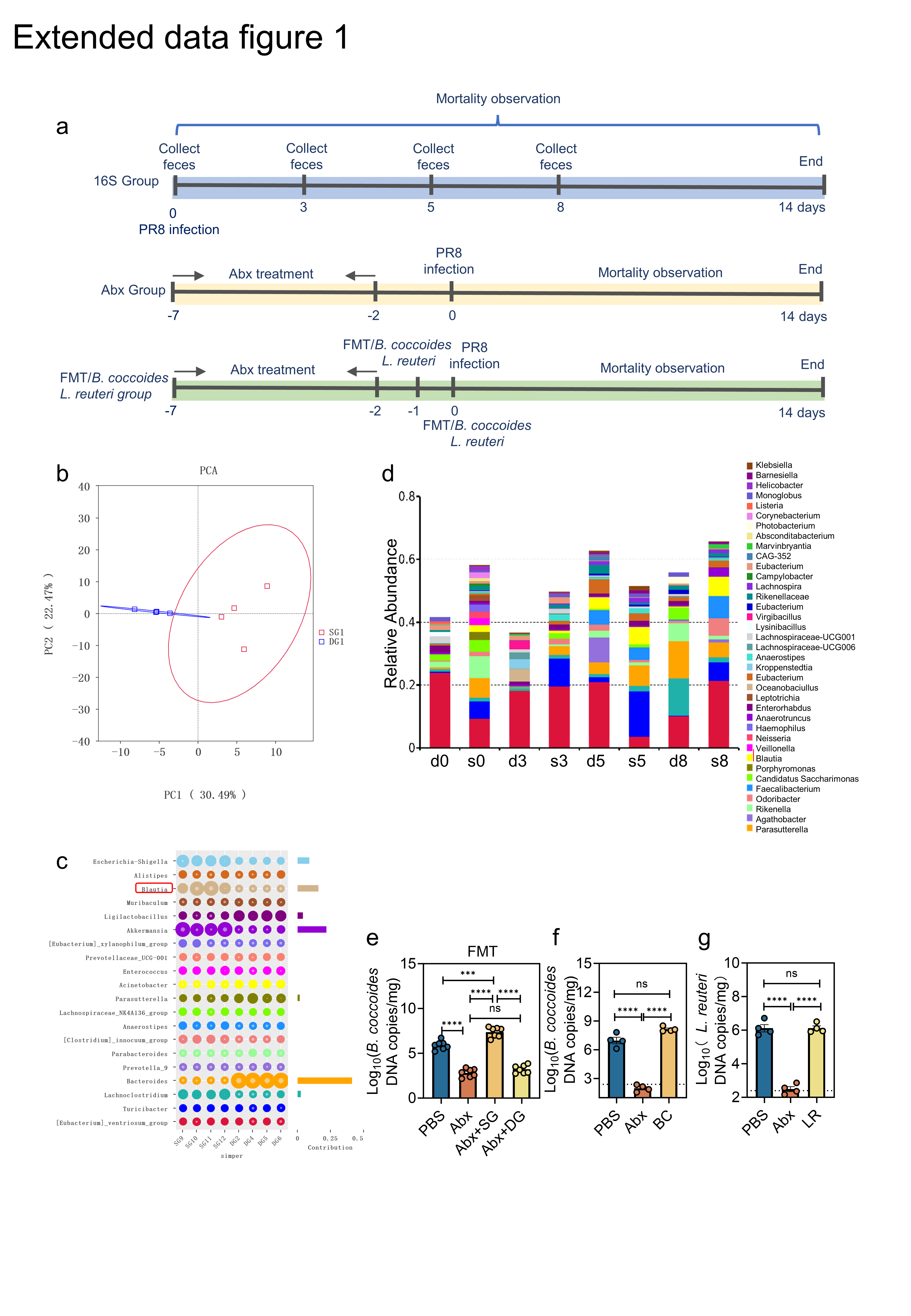

Supplement: Supplemental Material [file KGMI_A_2401649_SM8967.zip › Supplementary_files__41_ (1)/KGMI_A_2401649/Extended data figure 1.TIF]

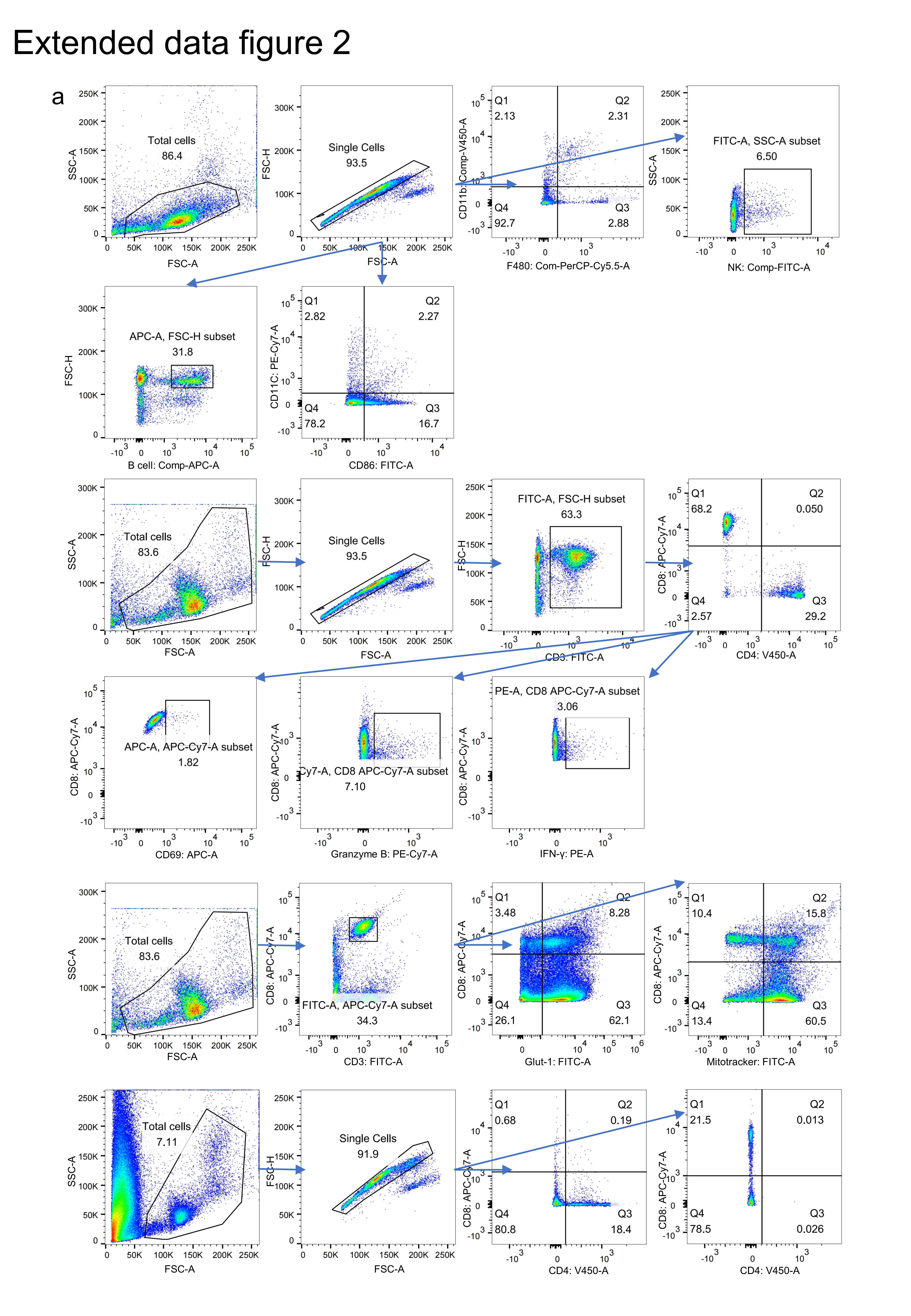

Supplement: Supplemental Material [file KGMI_A_2401649_SM8967.zip › Supplementary_files__41_ (1)/KGMI_A_2401649/Extended data figure 2-1.TIF]

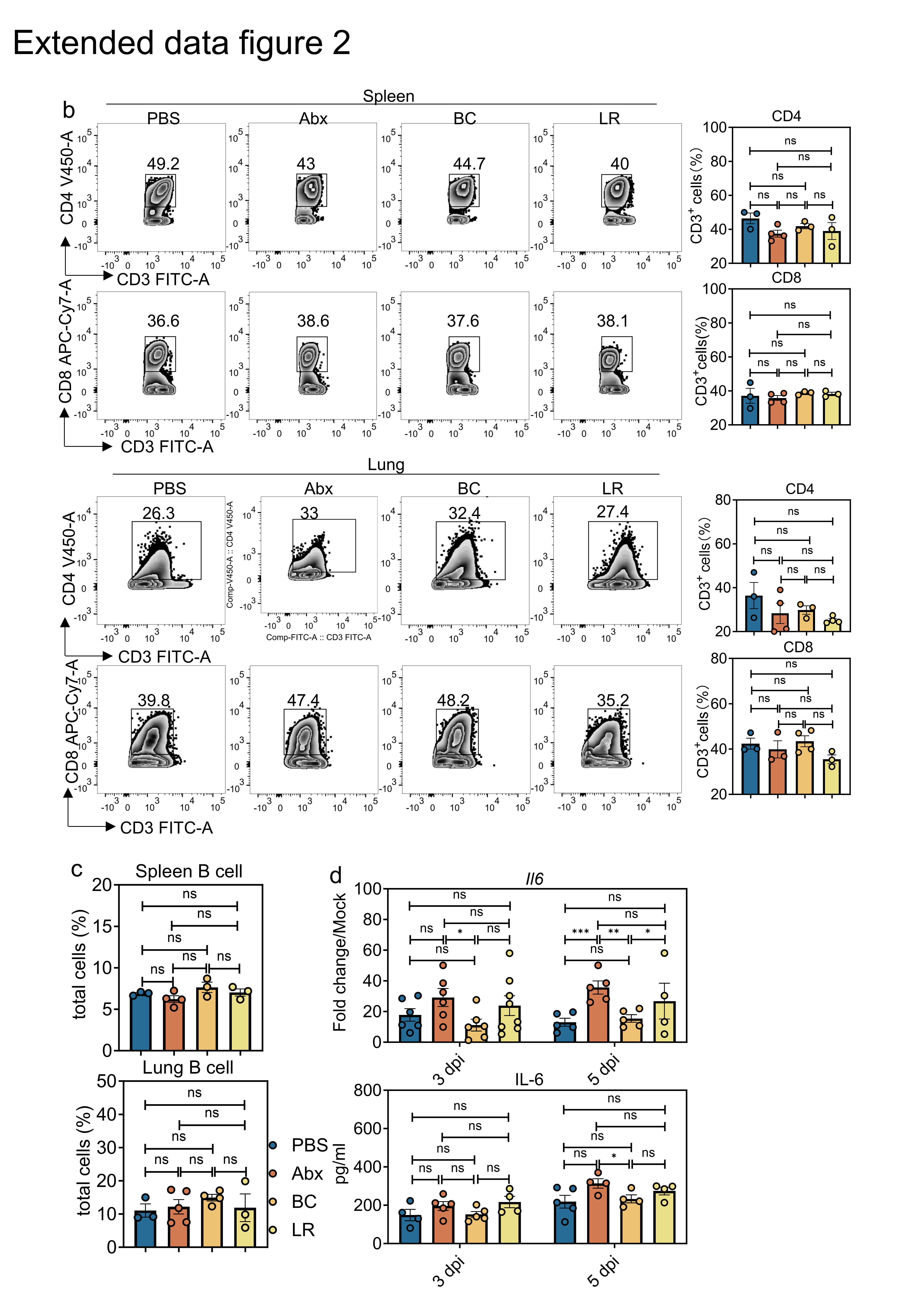

Supplement: Supplemental Material [file KGMI_A_2401649_SM8967.zip › Supplementary_files__41_ (1)/KGMI_A_2401649/Extended data figure 2-2.TIF]

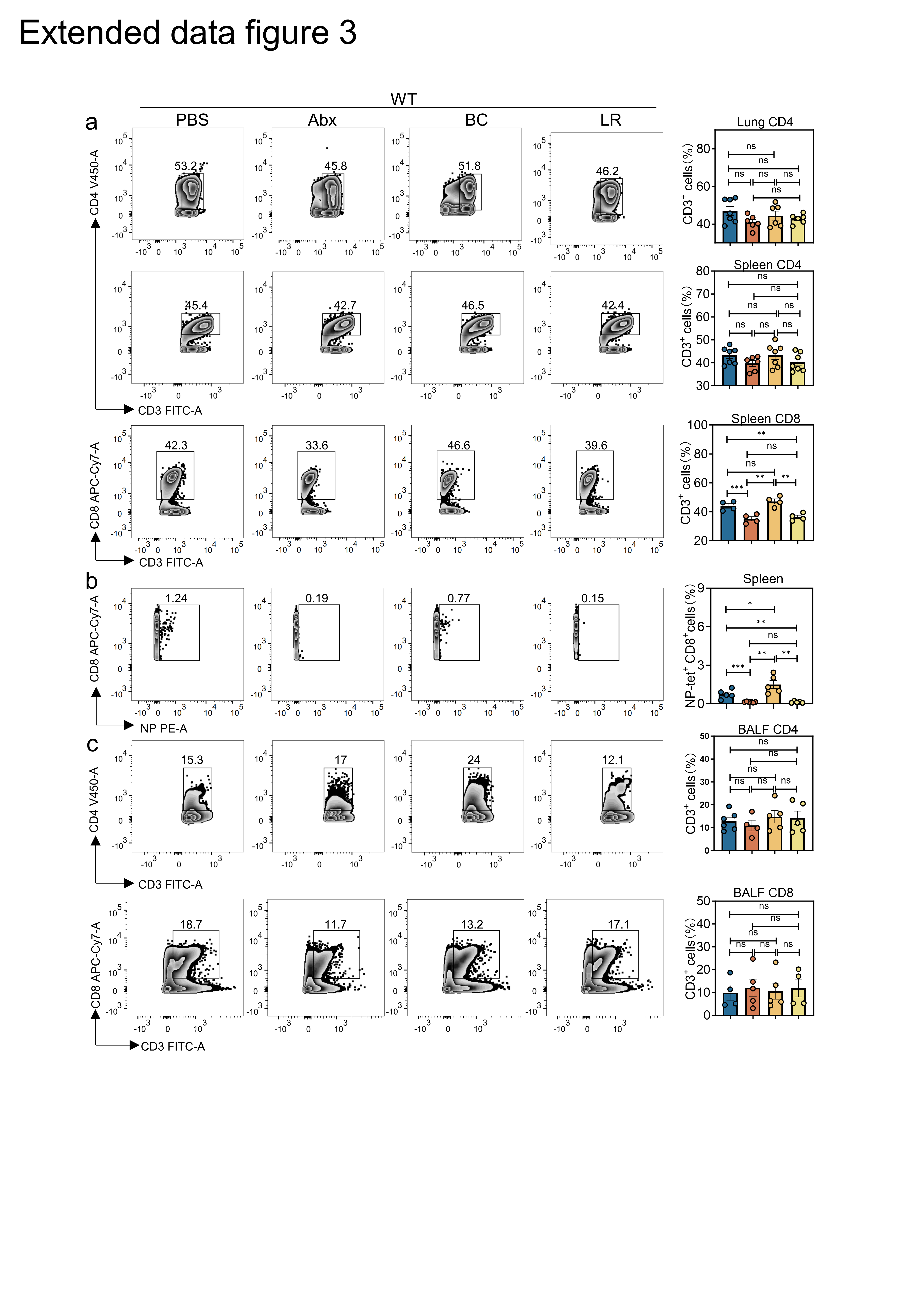

Supplement: Supplemental Material [file KGMI_A_2401649_SM8967.zip › Supplementary_files__41_ (1)/KGMI_A_2401649/Extended data figure 3-1.TIF]

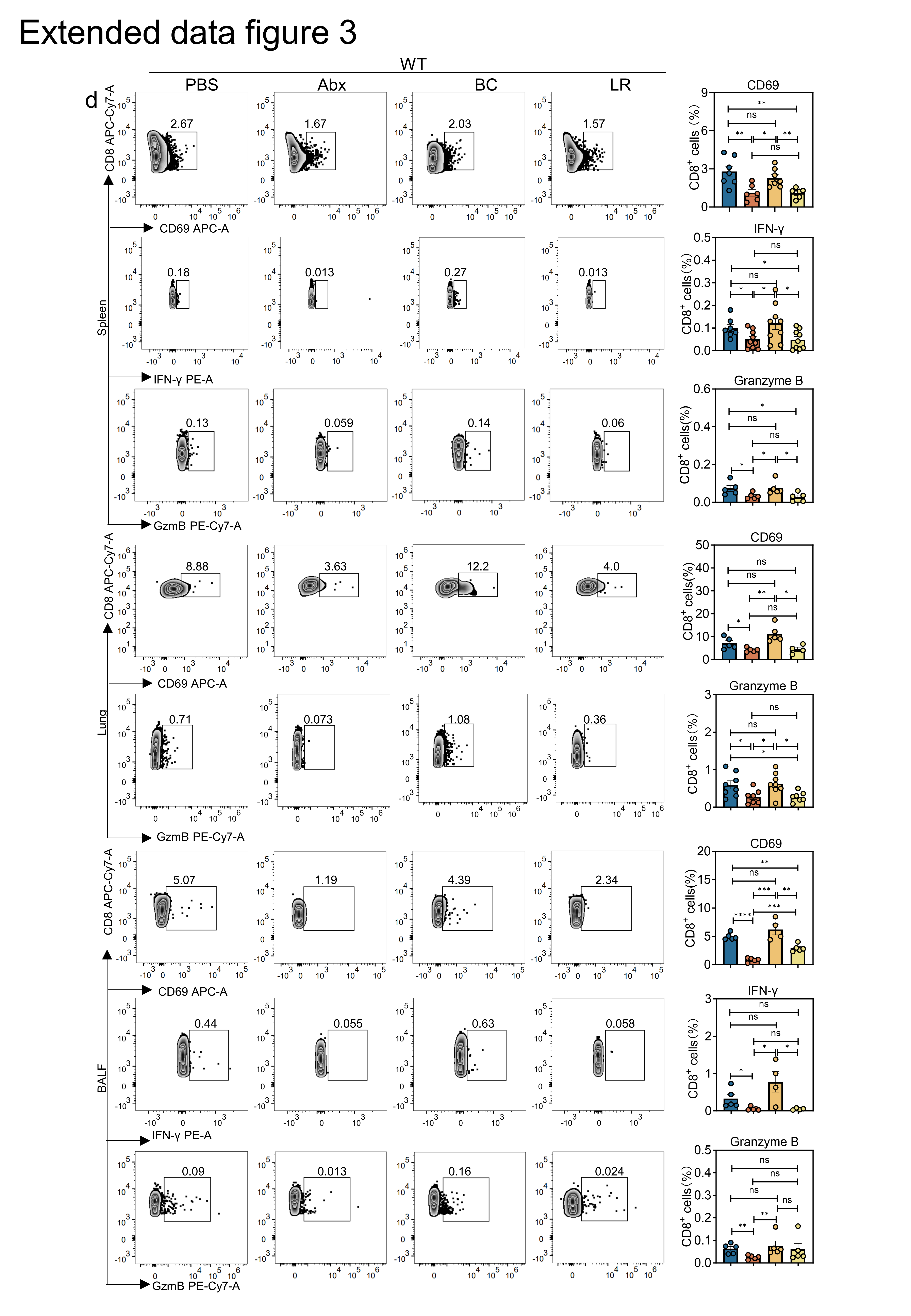

Supplement: Supplemental Material [file KGMI_A_2401649_SM8967.zip › Supplementary_files__41_ (1)/KGMI_A_2401649/Extended data figure 3-2.TIF]

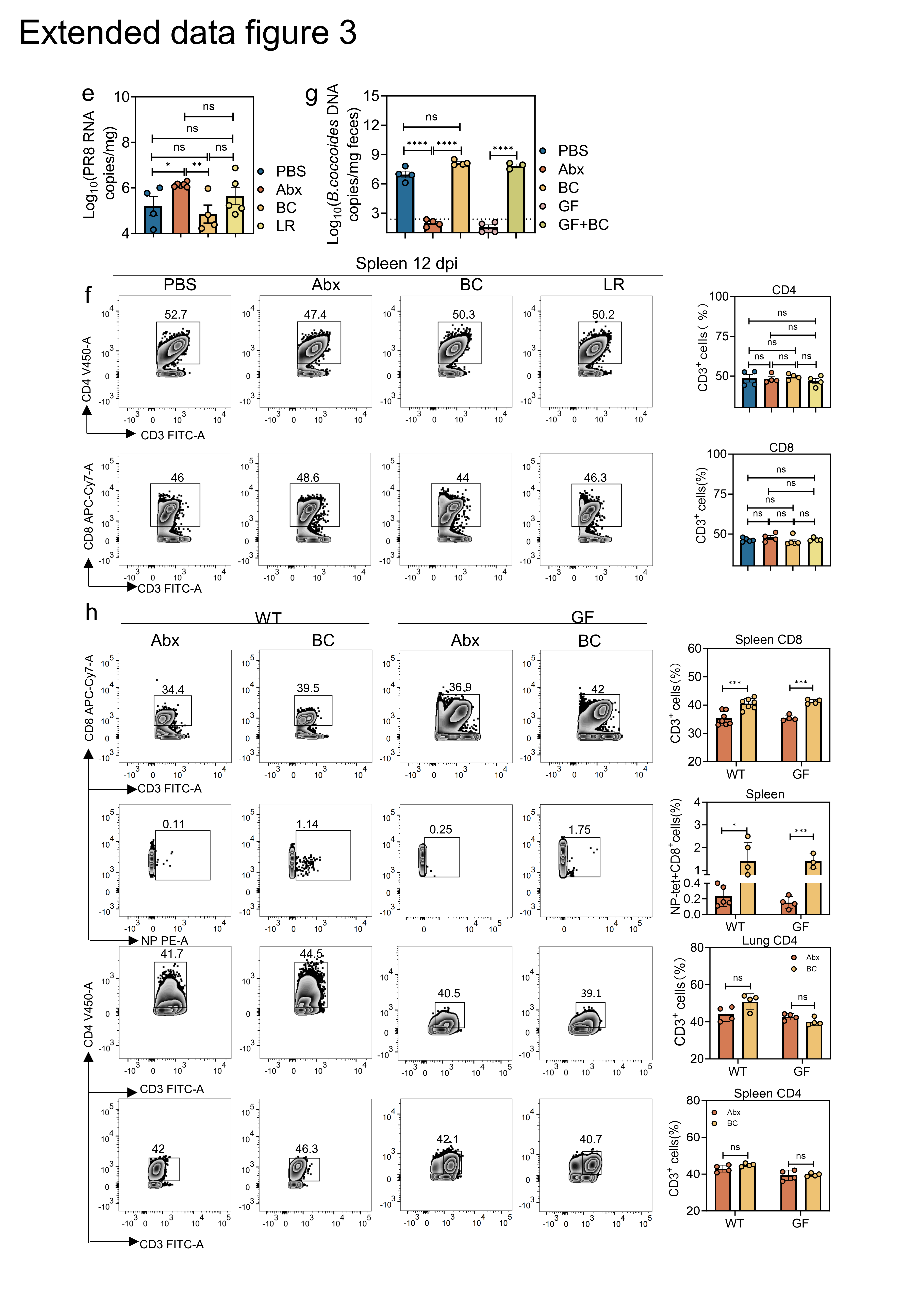

Supplement: Supplemental Material [file KGMI_A_2401649_SM8967.zip › Supplementary_files__41_ (1)/KGMI_A_2401649/Extended data figure 3-3.TIF]

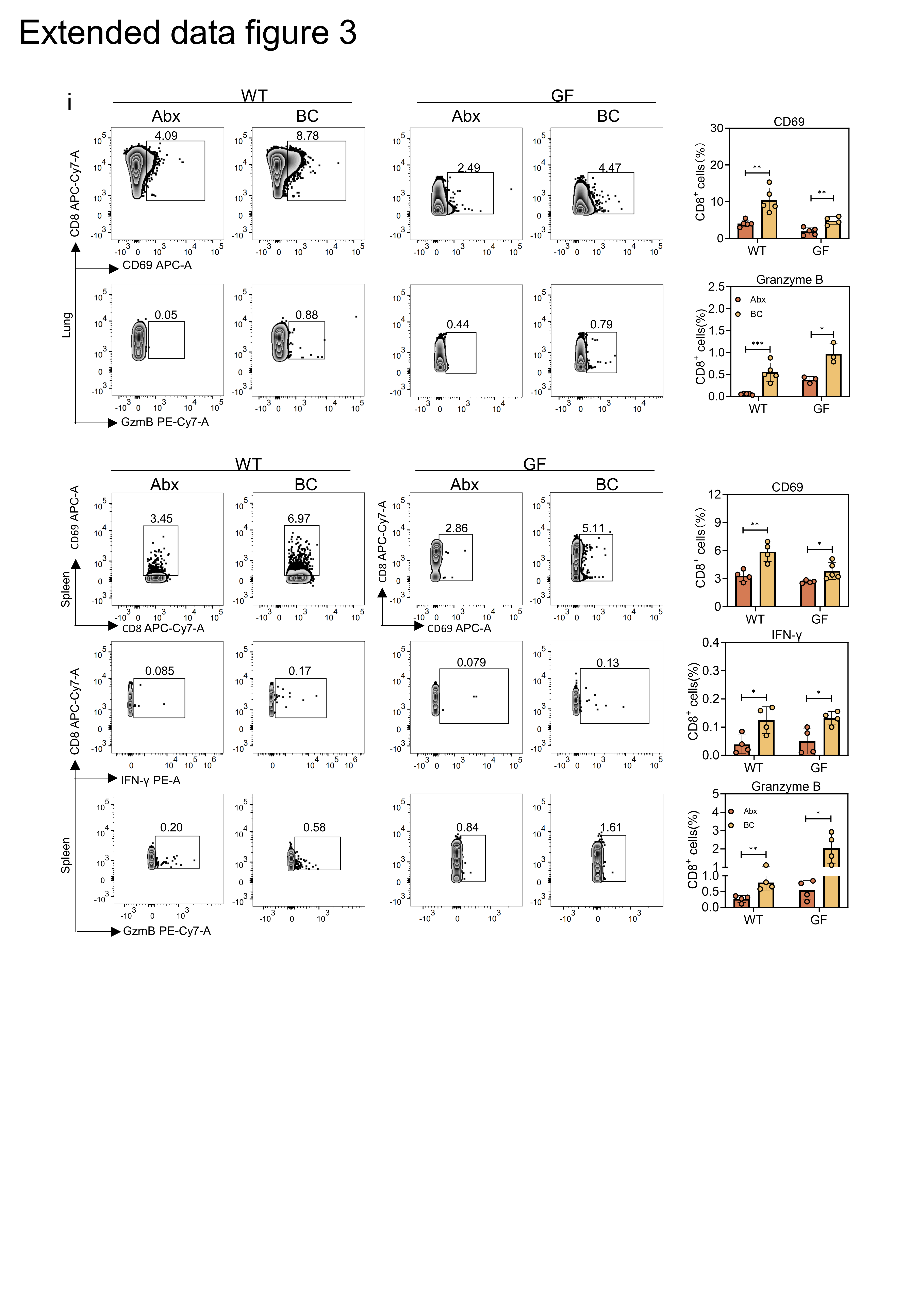

Supplement: Supplemental Material [file KGMI_A_2401649_SM8967.zip › Supplementary_files__41_ (1)/KGMI_A_2401649/Extended data figure 3-4.TIF]

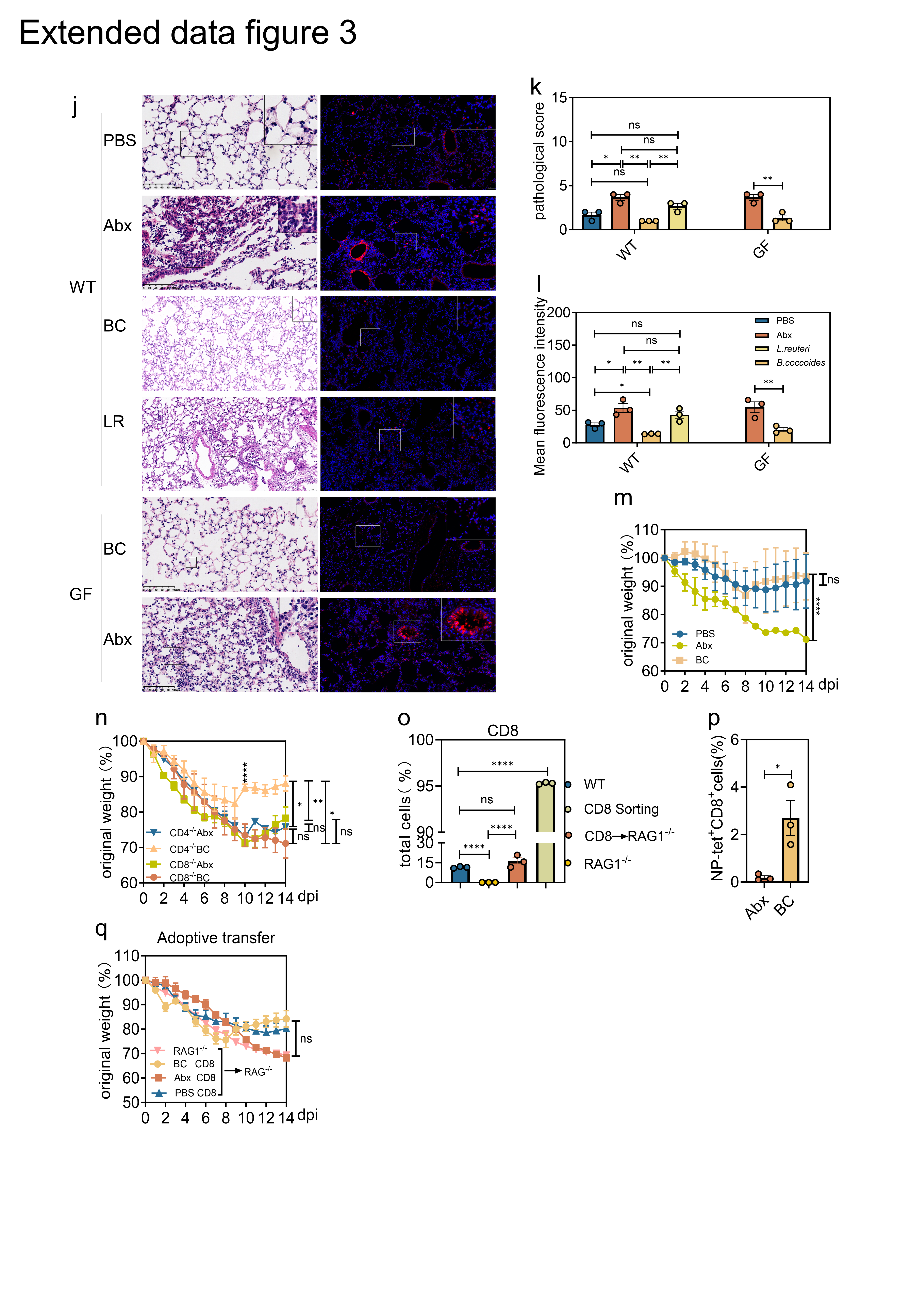

Supplement: Supplemental Material [file KGMI_A_2401649_SM8967.zip › Supplementary_files__41_ (1)/KGMI_A_2401649/Extended data figure 3-5.TIF]

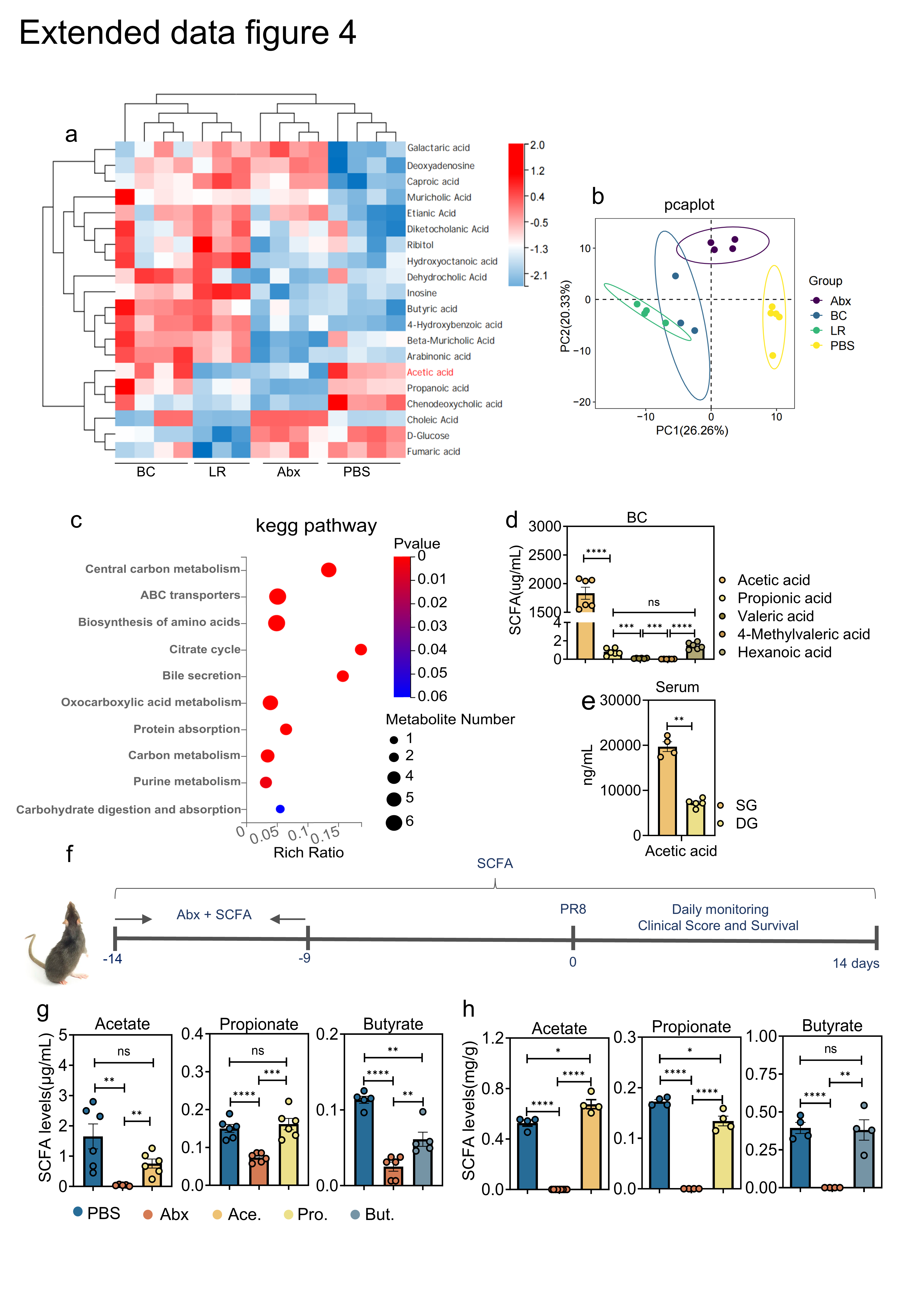

Supplement: Supplemental Material [file KGMI_A_2401649_SM8967.zip › Supplementary_files__41_ (1)/KGMI_A_2401649/Extended data figure 4-1.TIF]

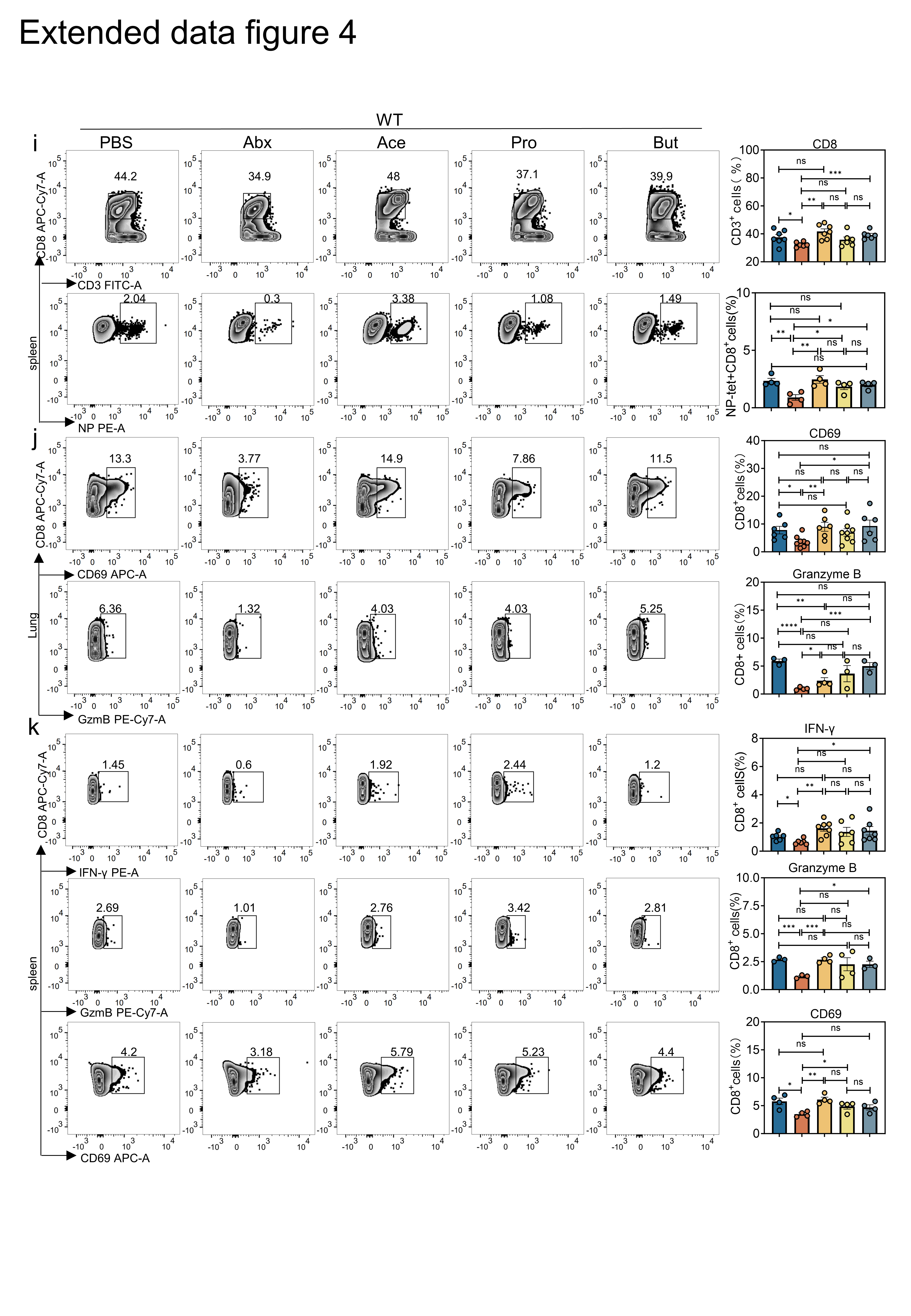

Supplement: Supplemental Material [file KGMI_A_2401649_SM8967.zip › Supplementary_files__41_ (1)/KGMI_A_2401649/Extended data figure 4-2.TIF]

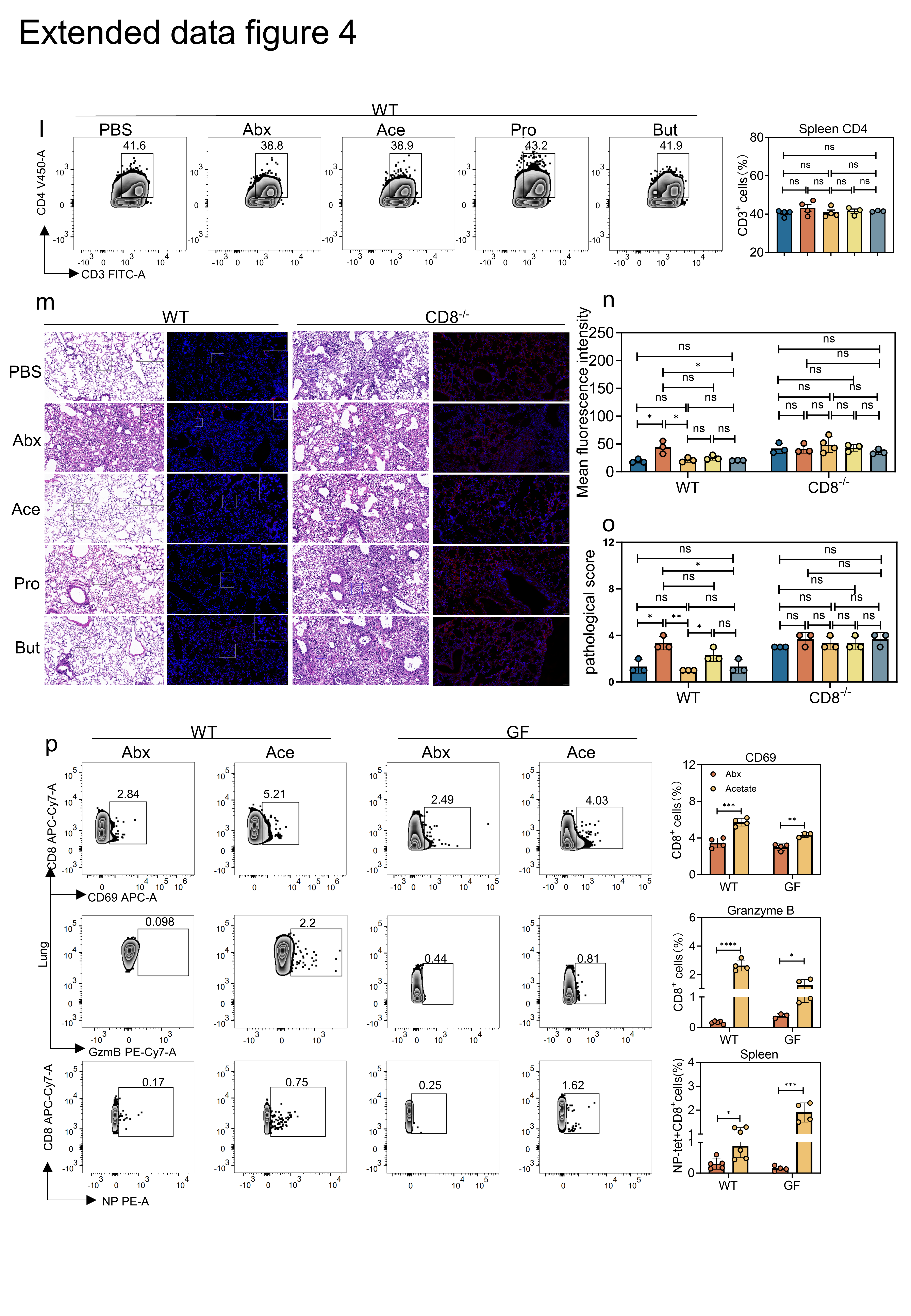

Supplement: Supplemental Material [file KGMI_A_2401649_SM8967.zip › Supplementary_files__41_ (1)/KGMI_A_2401649/Extended data figure 4-3.TIF]

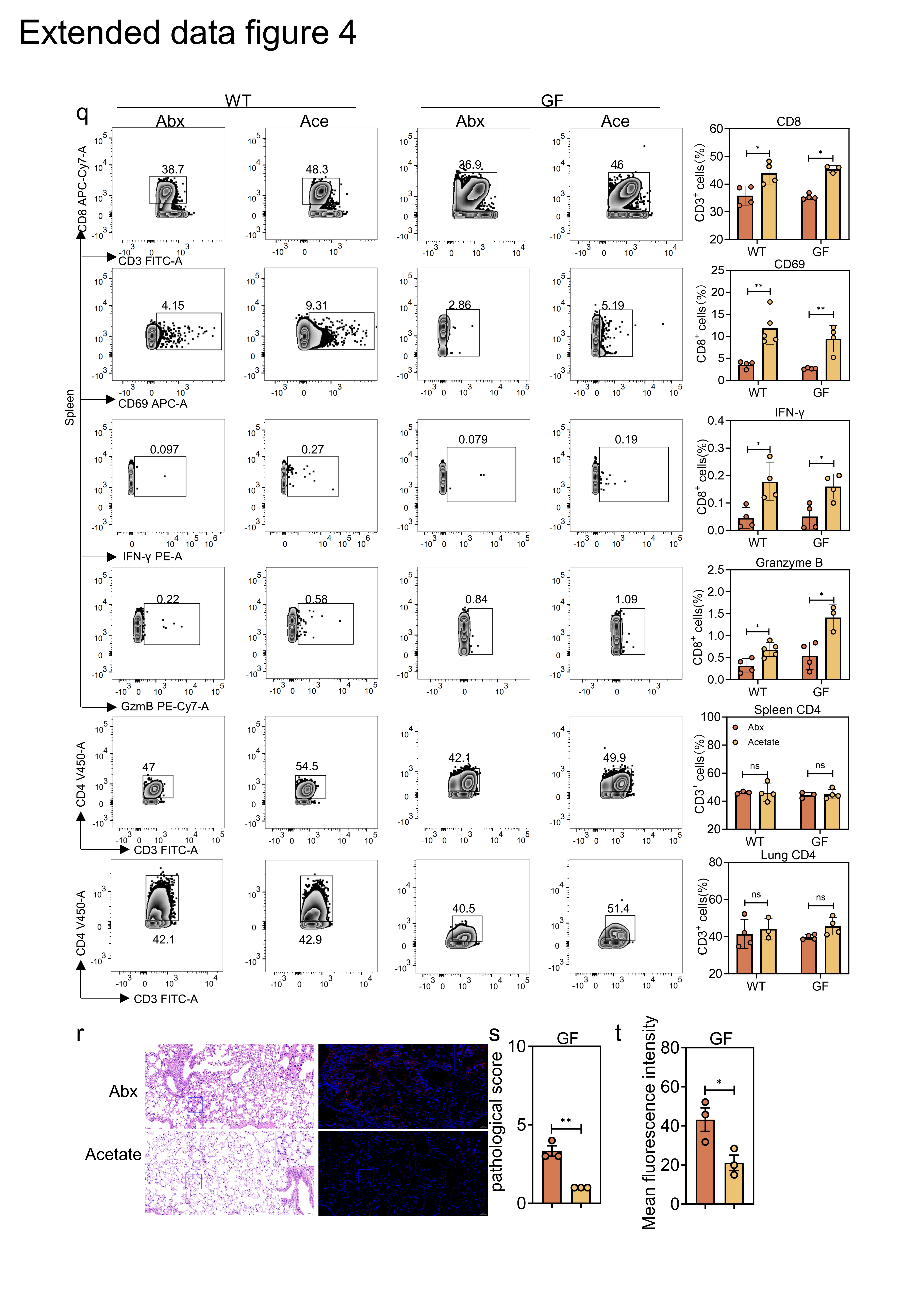

Supplement: Supplemental Material [file KGMI_A_2401649_SM8967.zip › Supplementary_files__41_ (1)/KGMI_A_2401649/Extended data figure 4-4.TIF]

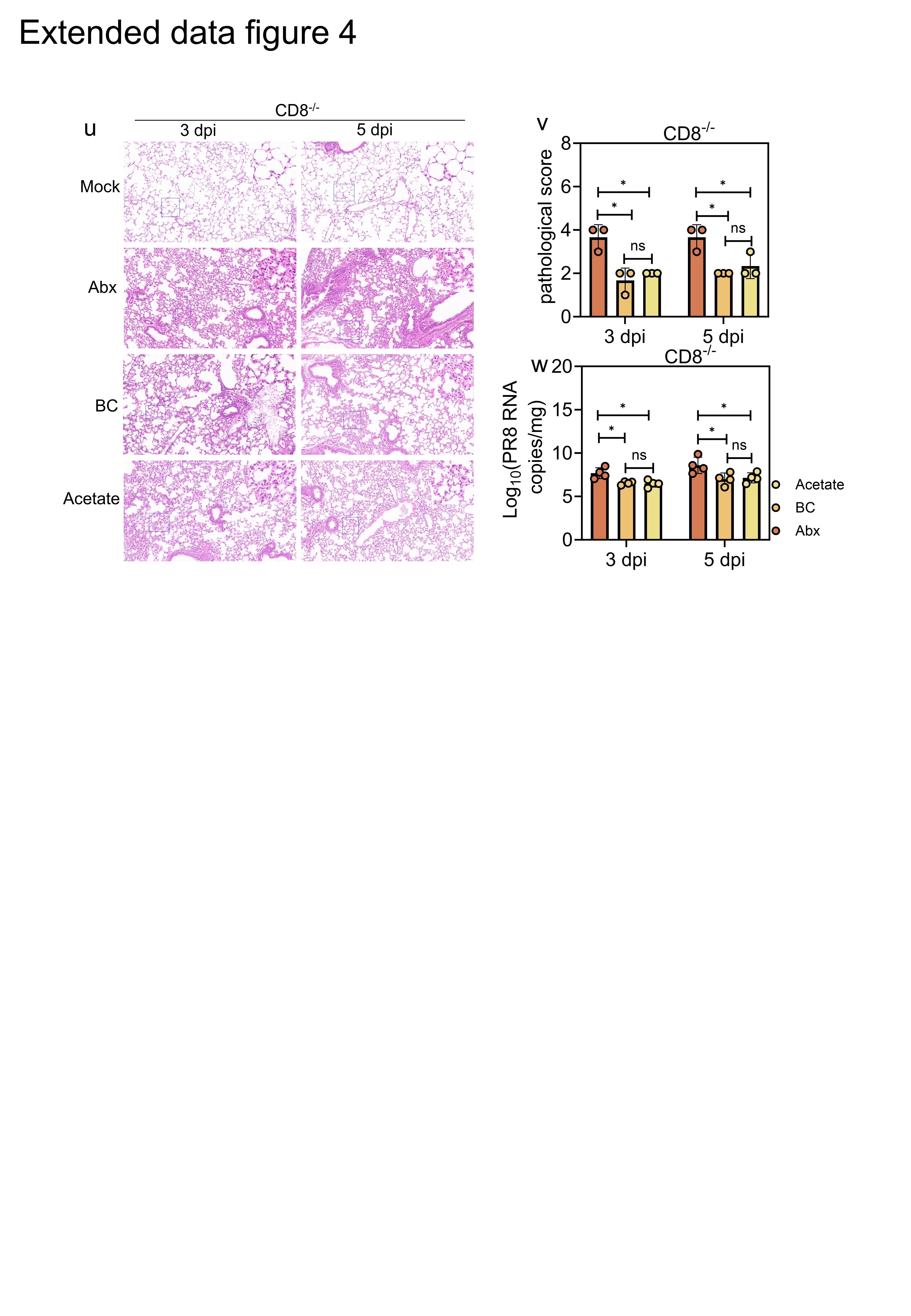

Supplement: Supplemental Material [file KGMI_A_2401649_SM8967.zip › Supplementary_files__41_ (1)/KGMI_A_2401649/Extended data figure 4-5.TIF]

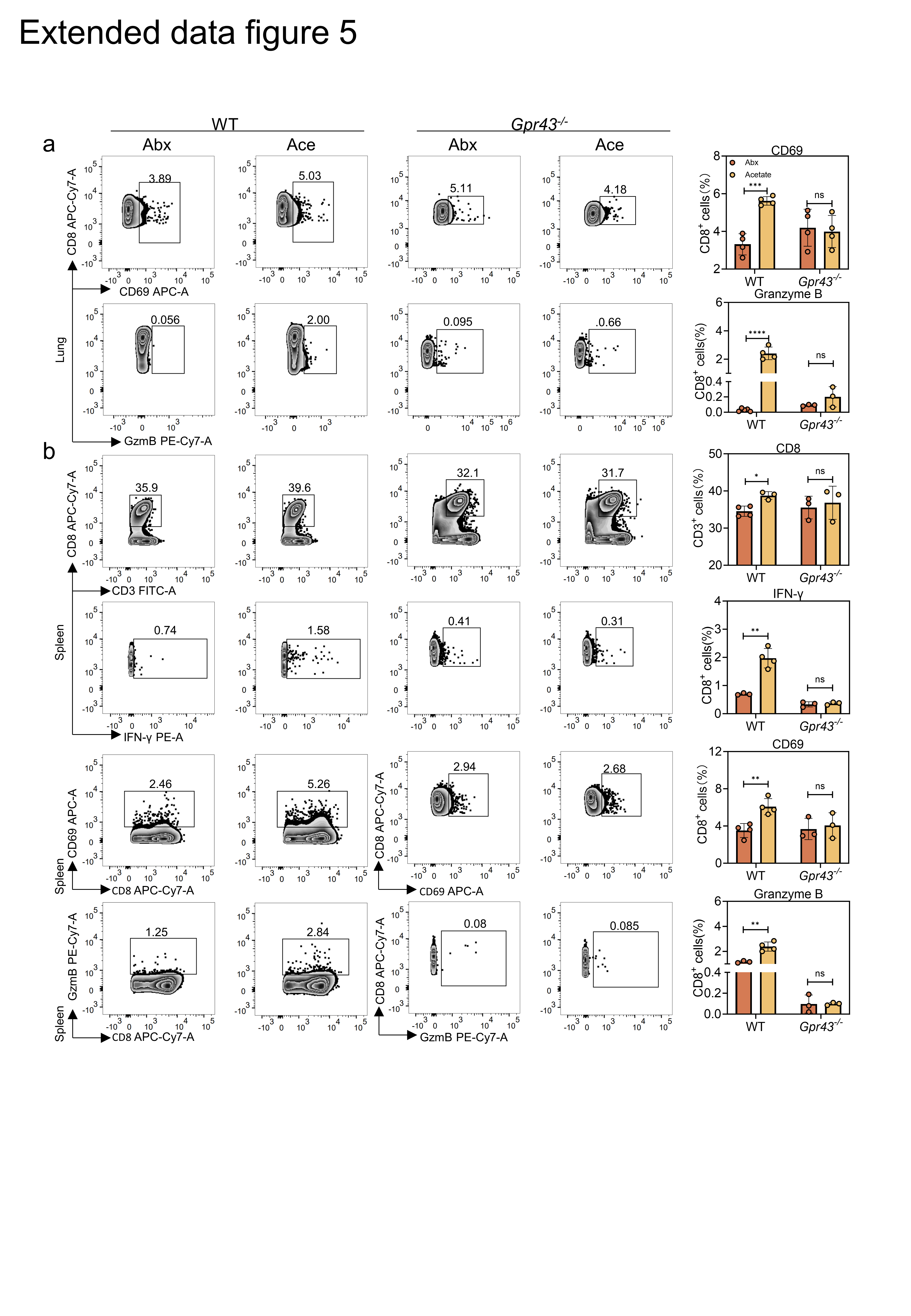

Supplement: Supplemental Material [file KGMI_A_2401649_SM8967.zip › Supplementary_files__41_ (1)/KGMI_A_2401649/Extended data figure 5-1.TIF]

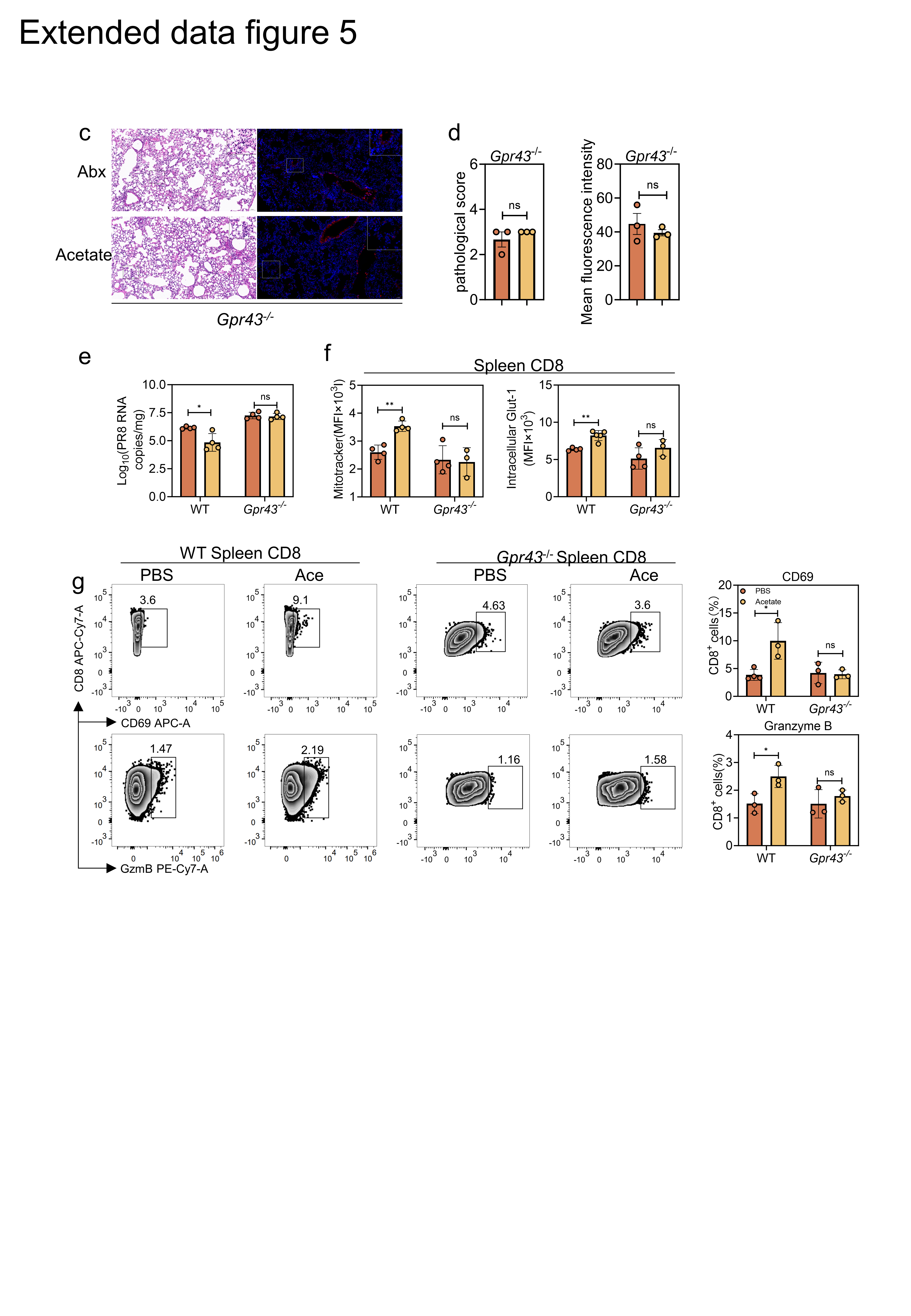

Supplement: Supplemental Material [file KGMI_A_2401649_SM8967.zip › Supplementary_files__41_ (1)/KGMI_A_2401649/Extended data figure 5-2.TIF]

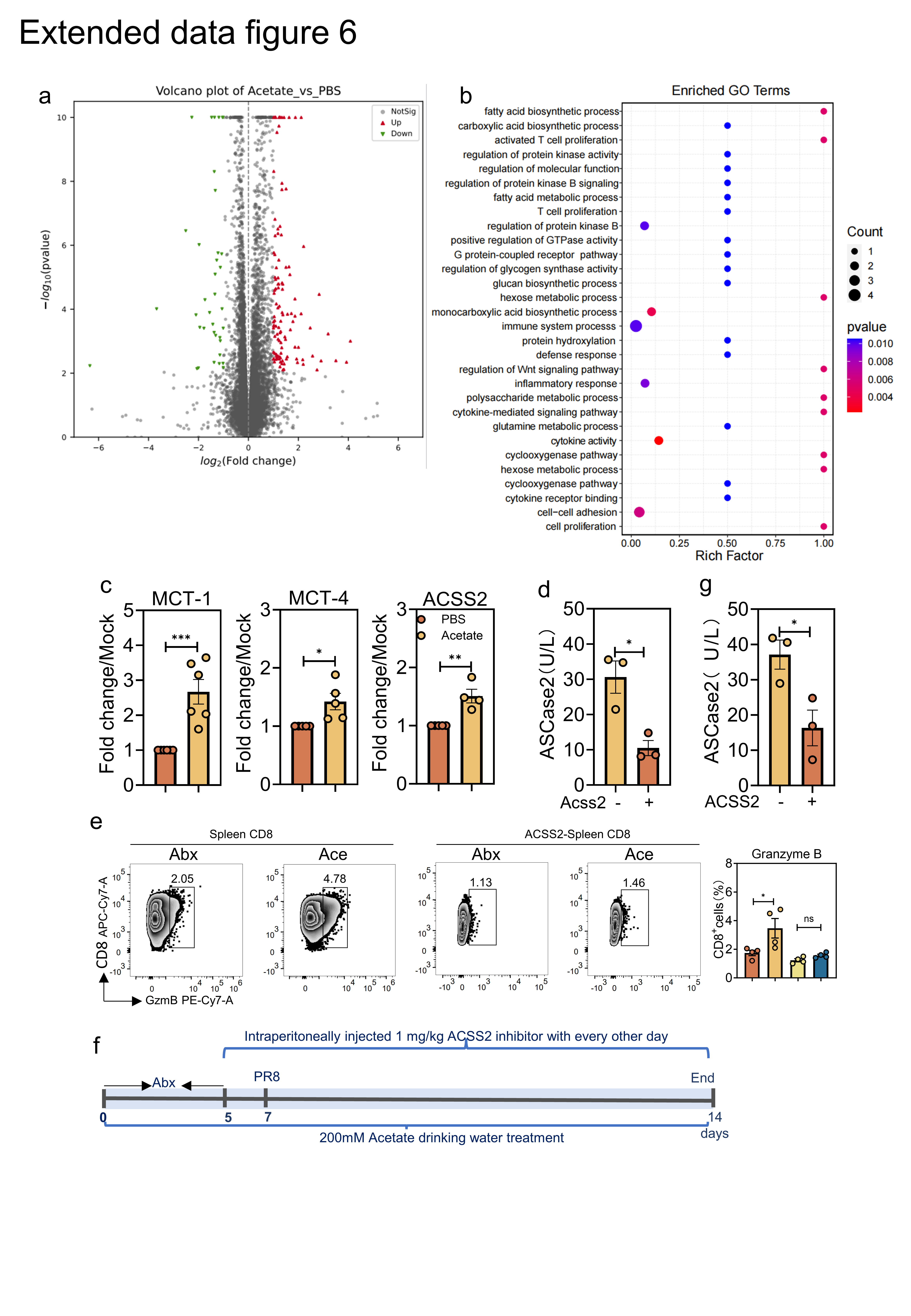

Supplement: Supplemental Material [file KGMI_A_2401649_SM8967.zip › Supplementary_files__41_ (1)/KGMI_A_2401649/Extended data figure 6-1.TIF]

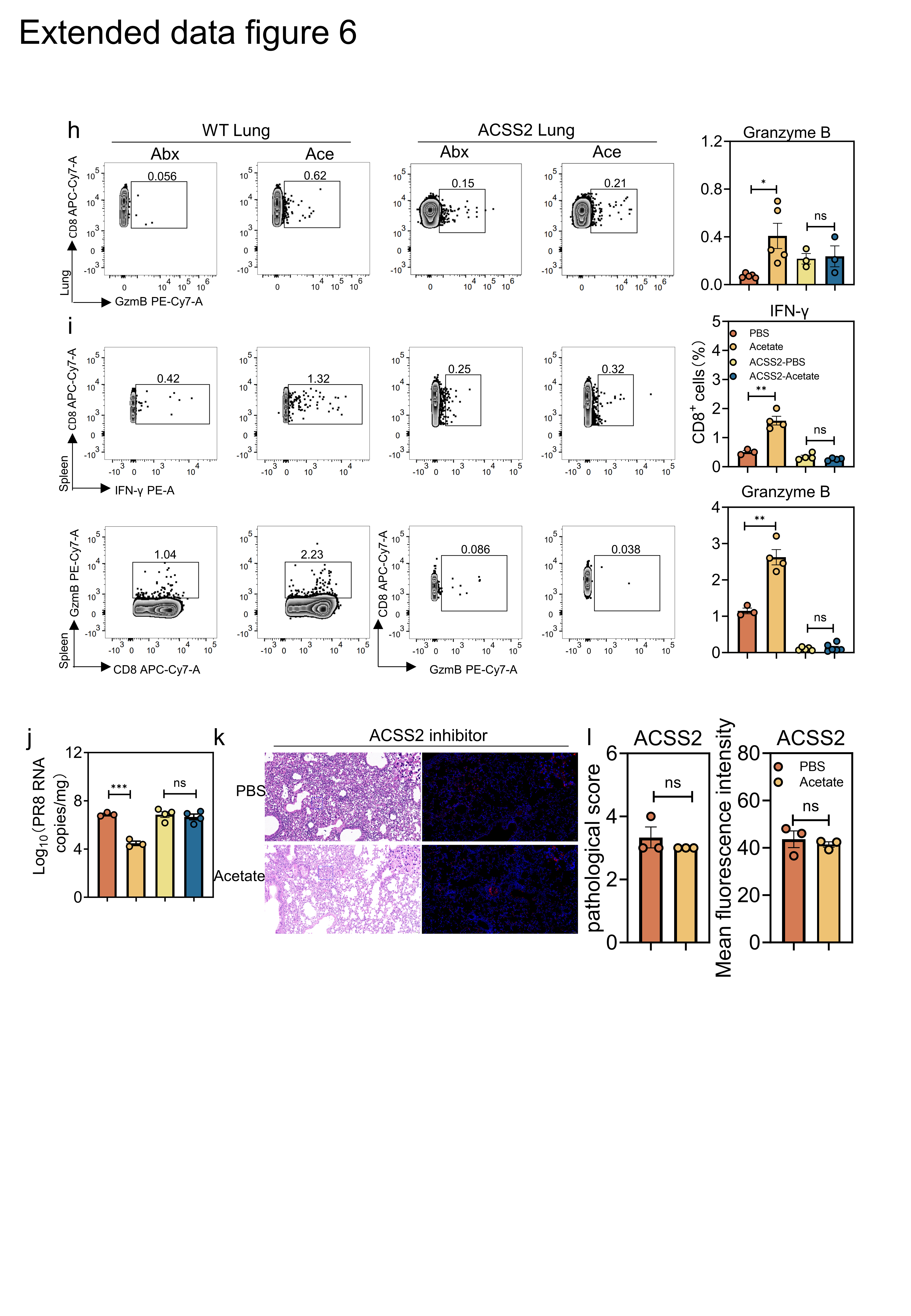

Supplement: Supplemental Material [file KGMI_A_2401649_SM8967.zip › Supplementary_files__41_ (1)/KGMI_A_2401649/Extended data figure 6-2.TIF]
